# Effect of Reverberation on Neural Responses to Natural Speech in Rabbit Auditory Midbrain: No Evidence for a Neural Dereverberation Mechanism

**DOI:** 10.1523/ENEURO.0447-22.2023

**Published:** 2023-05-10

**Authors:** Oded Barzelay, Stephen David, Bertrand Delgutte

**Affiliations:** 1 Eaton-Peabody Laboratories, Massachusetts Eye and Ear, Boston, MA 02114-3096; 2 Department of Otolaryngology, Head and Neck Surgery, Harvard Medical School, Boston, MA 02115; 3 Oregon Research Hearing Center, Oregon Health and Science University, Portland, OR 97239-3098

## Abstract

Reverberation is ubiquitous in everyday acoustic environments. It degrades both binaural cues and the envelope modulations of sounds and thus can impair speech perception. Still, both humans and animals can accurately perceive reverberant stimuli in most everyday settings. Previous neurophysiological and perceptual studies have suggested the existence of neural mechanisms that partially compensate for the effects of reverberation. However, these studies were limited by their use of either highly simplified stimuli or rudimentary reverberation simulations. To further characterize how reverberant stimuli are processed by the auditory system, we recorded single-unit (SU) and multiunit (MU) activity from the inferior colliculus (IC) of unanesthetized rabbits in response to natural speech utterances presented with no reverberation (“dry”) and in various degrees of simulated reverberation (direct-to-reverberant energy ratios (DRRs) ranging from 9.4 to –8.2 dB). Linear stimulus reconstruction techniques (Mesgarani et al., 2009) were used to quantify the amount of speech information available in the responses of neural ensembles. We found that high-quality spectrogram reconstructions could be obtained for dry speech and in moderate reverberation from ensembles of 25 units. However, spectrogram reconstruction quality deteriorated in severe reverberation for both MUs and SUs such that the neural degradation paralleled the degradation in the stimulus spectrogram. Furthermore, spectrograms reconstructed from responses to reverberant stimuli resembled spectrograms of reverberant speech better than spectrograms of dry speech. Overall, the results provide no evidence for a dereverberation mechanism in neural responses from the rabbit IC when studied with linear reconstruction techniques.

## Significance Statement

Reverberation is an acoustic phenomenon that is present in most everyday settings. It degrades perceptually important modulations in human speech and animal vocalizations. Nonetheless, normal hearing humans and animals easily perceive acoustic stimuli in most reverberant settings. Previous work has suggested that the auditory system may possess neural mechanisms that compensate for the effects of reverberation, but these studies used highly simplified stimuli or reverberation simulations. In this work, we examined the effect of reverberation on the neural coding of natural speech in the inferior colliculus (IC), a key processing stage in the auditory system. We found that neural responses were robust to a moderate amount of reverberation but found no evidence for neural dereverberation mechanisms in severe reverberation.

## Introduction

Everyday speech communication, whether indoors or in nature, usually unfolds in reverberant environments (Richards and Wiley, 1980; Traer and Mcdermott, 2016). In such environments, a listener receives the sound from an acoustic source as the sum of a direct sound combined with its delayed reflections from neighboring surfaces. These reflections arrive at the receiver from different directions and with various delays, thereby distorting both binaural cues (Moncur and Dirks, 1967; Shinn-Cunningham and Kawakyu, 2003; Hartmann et al., 2005) and perceptually-important temporal amplitude modulations (AM) of the source sound (Houtgast and Steeneken, 1973, 1985). Normal hearing human listeners can accurately localize sound sources and understand speech in everyday, moderately reverberant settings (Sato et al., 2007; W. Yang and Bradley, 2009). However, reverberation can cause more severe perceptual degradations when combined with other factors such as hearing loss (Nabelek and Pickett, 1974; Gelfand and Hochberg, 1976; Duquesnoy and Plomp, 1980; Reinhart et al., 2016), aging (Nábĕlek and Robinson, 1982; Helfer, 1994; Shinn-Cunningham and Kawakyu, 2003), and the presence of noise or competing sounds (Helfer, 1994; Neuman et al., 2010).

This relative robustness of the auditory system to reverberation depends on its ability to preserve or reconstruct the temporal AMs of the stimuli (Houtgast and Steeneken, 1973, 1985; Shannon et al., 1995). Indeed, psychophysical experiments offer some evidence for such a mechanism (Watkins, 2005; Zahorik et al., 2011), which suggests in turn a neural mechanism of compensation. On the physiological side, experiments on the cochlear nucleus of anesthetized guinea pigs (Sayles and Winter, 2008; Sayles et al., 2015) showed that the temporal coding of the fundamental frequency (F0) of harmonic complex tones is robust to reverberation for static F0s, but not for temporally modulated F0s. In the auditory midbrain of unanesthetized rabbits, reverberation was found to degrade the temporal coding of AM, as expected from the reduction in stimulus AM, but the amount of degradation in AM coding was not as large in the responses of most neurons as in the stimulus waveform, which could be interpreted as a form of reverberation compensation (Devore and Delgutte, 2010; Kuwada et al., 2014; Slama and Delgutte, 2015). These results were based on sinusoidally AM noise stimuli that lack the spectro-temporal complexity of real speech signals (Singh and Theunissen, 2003).

At a higher level in the auditory pathway, Mesgarani et al. (2014) measured responses to human speech and ferret vocalizations from the primary auditory cortex (A1) of awake ferrets in the presence of strong reverberation, albeit using a simplified reverberant impulse response. Using the optimal linear spectrogram reconstruction method (Bialek et al., 1991; Stanley et al., 1999; Mesgarani et al., 2009) to quantify the stimulus information available in ensemble neural responses, they showed that the reconstructed spectrograms resembled the “dry” stimulus spectrograms (stimuli without reverberation) more than the reverberant stimulus spectrograms. Their results provide support for a compensation process in the neural code of stimuli in A1 or earlier along the ascending auditory pathway. Recently, Ivanov et al. (2022) showed that neurons in the auditory cortex of anesthetized ferrets adapt to the reverberant environment by increasing the latency of the inhibitory components of their spectro-temporal receptive field (STRF) when the amount of reverberation increases. They further showed that this observation is consistent with predictions of a normative linear model for dereverberation that optimally reconstructs dry spectrograms from reverberant spectrograms.

In this study, we tested whether a dereverberation process already exists at the level of the inferior colliculus (IC) in the auditory midbrain. We used natural speech utterances as stimuli combined with a realistic, binaural reverberation simulation. We recorded from both single units (SUs) and multiunits (MUs) in the IC of unanesthetized rabbits under various degrees of reverberation. To quantify the amount of information about the stimulus utterances for the various reverberation conditions, we used the optimal spectrogram reconstruction method and compared spectrograms reconstructed from ensemble neural activity to both dry and reverberant stimulus spectrograms. High-quality spectrogram reconstructions were obtained for dry speech. However, reconstruction quality for reverberant stimuli degraded with increasing reverberation when assessed against the dry stimulus, and this degradation in the quality of spectrogram reconstruction paralleled the degradation in the stimulus spectrograms. Thus, we found no evidence for a reverberation compensation mechanism in the IC of unanesthetized rabbits when studied with speech utterances and linear stimulus reconstruction techniques.

## Materials and Methods

### Animal preparation

Two females and one male adult Dutch-belted rabbits were used for the neurophysiological experiments. All procedures were approved by the animal care and Use Committee of Massachusetts Eye and Ear. Procedures were adapted from Kuwada et al. (1987) and have been described previously (Su and Delgutte, 2019). Briefly, before the electrophysiological recordings, each rabbit underwent two aseptic surgeries while under anesthesia for the implantation of a head bar and a craniotomy, respectively. In both surgeries, anesthesia was induced with xylazine (6 mg/kg) and ketamine (35– 44 mg/kg) and was then maintained by either injection of one-third of the initial dose of xylazine and ketamine mix or facemask delivery of isoflurane gas mixed with oxygen (0.8 l/min, isoflurane concentration gradually increased to 2.5%).

In the first surgery for implantation of a head bar, the rabbit’s skull was exposed, and a brass bar and stainless-steel cylinder were fixed to the skull using dental cement. The head bar was used to fix the animal’s head during recording sessions and the cylinder helped to maintain an aseptic environment for a craniotomy. The cylinder was placed between the bregma and the lambdoid sutures, to enable access to the inferior colliculi (ICs) on both sides. After recovery and postoperative care of 7 d, the rabbit was trained to sit in the experimental apparatus with the head fixed during daily sessions for a week. At the end of the habituation period, a craniotomy of 2–3 mm in diameter was performed in a second procedure to enable access to the IC. Additionally, ear molds were made for each rabbit with vinyl polysiloxane material (Reprosil, Patterson Dental) to allow reproducible delivery of acoustic stimuli to the animal ears. Recording sessions began after 2–3 d of an additional recovery period. Throughout the recording period, auditory brain stem responses (ABRs) were measured in response to 100-μs clicks to verify normal hearing (threshold < 30 dB SPL). Over the course of the recording sessions (which could last for several months), additional surgeries were performed as needed to clean the exposed dura of scar tissue and, if needed, to slightly enlarge the craniotomy.

### Stimuli

**Figure 1.**
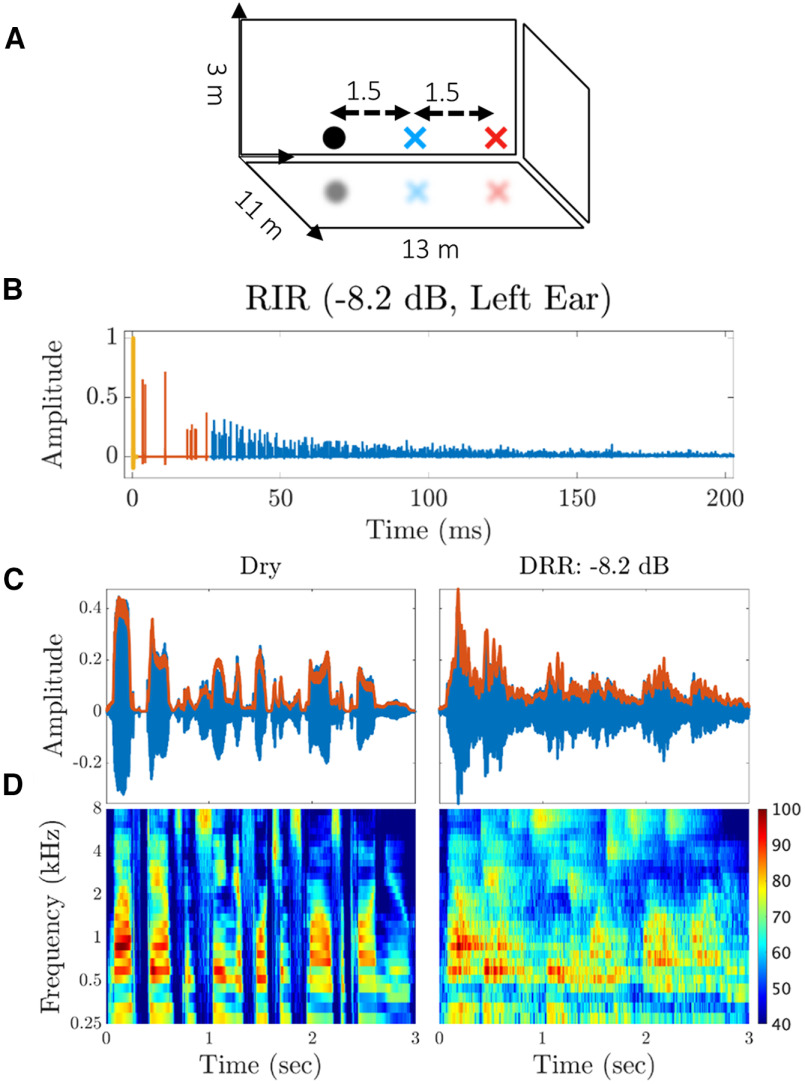
Effect of reverberation on a speech stimulus. ***A***, Dimensions of the virtual room (13 × 11 × 3 m) used to simulate binaural room impulse responses (BRIRs) by the room-image method. The source speaker was positioned at either 1.5 m (blue x) or 3.0 m (red x) in front of the receivers (0° azimuth). For each of these two source-to-listener distances, we also varied the wall absorption coefficients: 20% for a highly reverberant room, 80% for a mildly reverberant room, and 100% for no reverberation (also known as dry condition). Overall, we simulated five BRIRs, one dry condition, and four reverberant conditions with direct-to-reverberant energy ratios (DRRs) ranging from +9.4 to −8.2 dB. ***B***, The first 200 ms of an example BRIR for the most reverberant case (DRR = −8.2 dB, left ear). The BRIR is composed of the direct sound (yellow), individual early reflections (red), and overlapping late reflections (blue). ***C***, Waveform (blue) and broadband envelope (red) of the utterance “Laugh, dance, and sing if fortune smiles on you” pronounced by a female speaker for dry and highly reverberant conditions. ***D***, Spectrograms of the dry and reverberant utterances in ***C***. Each row in the spectrogram represents the bandpass Hilbert envelope of the stimulus with a center frequency on a log scale given on the *y*-axis. Adding reverberation smears the stimulus envelope, prolongs onsets, and offsets, and fills the silent intervals between sound segments. Speech was filtered through a logarithmically spaced gammatone filterbank that simulates the response of the auditory nerve (Patterson–Holdsworth ERB Filter Bank). The spectrograms contain 30 frequency channels with center frequencies ranging from 250 Hz to 8 kHz and a temporal sampling interval of 5 ms.

The virtual acoustic room technique was used to introduce reverberation into the stimuli by convolving binaural room impulse responses (BRIRs) with human speech, as was done for noise stimuli in previous papers from our laboratory (Devore et al., 2009; Slama and Delgutte, 2015). We used one speech stimulus without any reverberation (henceforth called “dry” stimulus) and four versions of this stimulus with various degrees of reverberations (Fig. 1; Table 1). The dry stimulus consisted of 12 utterances pronounced by six male and six female speakers that were randomly selected from the TIMIT corpus (Garofolo et al., 1993). The 12 utterances were concatenated into a 36-s signal. To create reverberant stimuli, the dry stimulus was convolved with a pair of binaural room impulse responses, one for each ear, and the output signal was truncated to 36 s (see below, Binaural room impulse responses). The order of the utterances within each of the 36-s stimuli for the dry and reverberant conditions was fixed throughout the experiments; however, the order of the presentation of these 36-s-long stimuli was randomized across dry and reverberant conditions for each recording. Each of the 36-s stimuli, for each reverberant condition, was repeated five times in a different random order. The dry stimulus was typically repeated another five times (10 times total) to obtain additional data to train the model used for spectrogram reconstruction (see Computational Modeling). Thus, the total duration of the speech stimuli played to each neuron was ∼15 min (∼5 × 5 × 36 s/60). For the analysis, we used only complete measurements (i.e., lasting for the entire 15 min).

**Table 1:** Virtual room parameters and reverberation metrics

Source-to- listener distance (m)	Wall absorption coefficient	Direct-to- reverberant energy ratio (DRR)	Reverberation time (RT_60_)
1.5 or 3.0	100%	Dry (Inf)	N/A
1.5	80%	9.4 dB	0.32 s
1.5	20%	−2.5 dB	2.03 s
3.0	80%	4.8 dB	0.32 s
3.0	20%	−8.2 dB	2.03 s

Binaural room impulse responses (BRIRs) were computed for each reverberant condition and for dry (i.e., no reverberation; 100% wall absorption coefficient). The table indicate the direct-to-reverberant energy ratio (DRR), which indicates the amount of reverberation in a stimulus, and the RT_60_, which is the duration over which the reflected sound decays by 60 dB from the beginning of the late reflection phase. Note that source-to-receiver distance and wall absorption coefficient affect two different aspects of reverberation.

#### Binaural room impulse responses

Binaural room impulse responses (BRIRs) were computed for each reverberant condition (Table 1) using the room-image method (Allen and Berkley, 1979; Shinn-Cunningham et al., 2001). Following Slama and Delgutte (2015), we simulated a rectangular virtual room (Fig. 1*A*) with dimensions appropriate for a classroom (11 × 13 × 3 m) and containing an acoustic source (speaker) and two-point receivers (the listener’s ears). The two receivers were located approximately (but not exactly) at the center of the room (4.7 × 5.4 × 1.4 m). The distance between the two receivers was set to 10.3 cm to create a maximum ITD of 300 μs typical for a rabbit (Day and Delgutte, 2013). For simplicity, the rabbit’s head in between the receivers was not explicitly modeled, so the simulated BRIRs did not include the effect of the acoustic head shadow. This means that only the effect of reverberation on interaural time differences (ITD) was simulated, but not the effect on interaural level differences.

Two parameters were used to manipulate the amount of reverberation in the BRIRs: the source-to-receiver distance and the wall absorption coefficients. The source was positioned either at 1.5 or 3.0 m and at $0^{0}$ azimuth relative to the receivers (Fig. 1*A*). The shorter distance (1.5 m) resulted in moderate reverberation and the longer distance (3.0 m) in stronger reverberation. We also varied the absorption coefficient of the virtual-room walls. We used three absorption coefficients for each distance: 100% absorption which corresponds to no reverberation (dry stimulus), 80% absorption which corresponds to mild reverberation, and 20% absorption which corresponds to strong reverberation. For simplicity, the absorption coefficients were assumed to be independent of frequency, unlike natural BRIRs, which show greater absorption at higher frequencies (Traer and Mcdermott, 2016).

As shown in Table 1, the source-to-receiver distance and wall absorption coefficient affect two different aspects of reverberation: the direct-to-reverberant energy ratio (DRR) and the reverberation time (RT_60_; see definitions below). Varying the distance affects the DRR but not the RT_60_. In contrast, varying the absorption coefficient changes both the DRR and the RT_60_ (Table 1), but the effect on DRR is indirect.

#### Direct-to-reverberant energy ratio

We used the direct-to-reverberant energy ratio (DRR) to quantify the amount of reverberation in each stimulus. The DRR is the ratio of the energy in the direct sound to the energy of all the reflections, expressed in decibels. It was computed for each ear, and then averaged over the two ears. Our stimuli had DRRs ranging from infinity (dry condition) down to −8.2 dB for the most reverberant condition (20% absorption and source at 3.0 m). Figure 1*B* shows the first 200 ms of the left-ear BRIR for the most reverberant condition ( DRR=−8.2dB). The direct sound (first yellow peak) is followed by individually resolved early reflections (red peaks) and finally by overlapping late reflections (blue peaks) forming the reverberant tail of the BRIR.

#### Reverberation time

Reverberation time (RT_60_) is another metric, in addition to the DRR, that is commonly used to quantify the amount of reverberation. RT_60_ is the duration over which the reflected sound decays by 60 dB from the beginning of the late reflection phase (Fig. 1*B*, blue reflections). Speech intelligibility is known to degrade with Increased RT_60_ (Gelfand and Hochberg, 1976; Zahorik and Brandewie, 2016).

In a room of a given size, RT_60_ is affected mainly by the acoustic properties of the walls, floor, and ceiling; these properties were adjusted in our virtual room simulation by changing the acoustic absorption coefficients. We calculated RT_60_ directly from the room impulse response (RIR) for each reverberant condition (Table 1). Specifically, we computed the “peak envelope” (MATLAB’s “envelope” command) over successive 10-ms segments of the RIR; then we applied a moving-average filter (20-ms integration time), and finally, we performed a linear regression over the tail of the response (RIR expressed in dB) for the RIR time series.

### Electrophysiology

Recording sessions were performed in a double-walled, electrically shielded, and sound-treated chamber. During a session, the rabbit was restrained in a spandex sleeve and its head fixed through the mounted head bar. Each recording session lasted for up to 2.5 h throughout which the animal was monitored by a closed-circuit video. The recording session was terminated immediately if the rabbit showed any sign of distress or moved excessively. Sound stimuli were delivered through custom-made ear inserts made of vinyl polysiloxane impression material (Dentsply International Reprocil). Stimuli were generated on a digital computer in MATLAB, converted into analog signals using a 24-bit digital-to-analog converter (National Instruments PXI-4461), and delivered to loudspeakers (Beyer-Dynamic DT-48) connected by flexible tubes to the rabbit’s ear inserts. At the beginning of each session, the acoustic pressure inside the ear canal was measured using a probe-tube microphone (Etymotic ER-7C) in response to a broadband chirp stimulus. Using this calibration, an inverse digital filter was then created and applied to all stimuli to yield a flat transfer function over the frequency range from 50 Hz to 18 kHz.

For the neural recordings, we used polyimide-insulated, platinum-iridium linear microelectrode arrays (LMAs; MicroProbes) with six to eight contacts that were spaced 150 μm apart and impedances of 0.2–1.0 Ω in each of the recording channels. A remote-controlled hydraulic micro-positioner (David Kopf Instruments model 650) was used to advance the LMA through the occipital cortex and into the IC. The signals recorded from the microelectrode array were amplified, bandpass filtered (0.3–5 kHz, Plexon PBX2), and sampled at 100 kHz (National Instruments, PXI-6123).

The IC units were identified by audiovisual cues of synchronization to a search stimulus consisting of broadband noise bursts presented diotically (200 ms on, 300 ms off) at 60 dB SPL. Upon identifying the IC with single units (see below), the set of five speech stimuli (the dry stimulus along with the four reverberant stimuli) were played continuously and responses were recorded.

To set the amplitude for each of these time-varying signals, we calculated a set of root mean square (RMS) amplitudes over a sliding window of 40 ms. Out of these values, we picked the 95th percentile as the signal’s RMS amplitude. We then averaged that RMS value over the left and right audio channels. This operation was repeated for each stimulus and each reverberation condition. Finally, we converted these RMS values to dB SPL. The stimuli were played over the earphones with amplitudes in the range of 60–70 dB SPL.

The order of presentation of the five reverberant conditions was randomized, as stated above; each reverberant condition was repeated five times and the dry stimulus was usually repeated 10 times. Neural responses (single-unit and multiunit responses) to all stimulus trials were combined offline into a single average response for each stimulus condition (see Signal processing of single and multiunit responses).

#### Pure-tone frequency response areas

In 65 out of the 103 single units studied, we measured the frequency response area (FRA) to characterize the neurons’ pure-tone frequency tuning. To measure an FRA, a series of 100-ms pure-tone bursts separated by 100 ms of silence were presented. The frequencies of the tones ranged from 200 Hz to 17 kHz in 0.25-octave steps, and the amplitudes ranged from 5 to 70 dB SPL; each tone was repeated 3 times in random order. The resulting FRA is visualized as a two-dimensional heatmap that shows firing rate as a function of pure tone frequency and amplitude (Fig. 3).

### Signal processing of single and multiunit responses

Signals from the LMA that contained single units (SU) with clear and stable spike shapes at one or more recording sites were identified. Spikes were detected whenever their amplitude crossed both a high and a low threshold that were set manually above and below the noise floor, respectively. Specifically, for a signal to qualify as a spike, the high threshold and then the low threshold had to be crossed in that order. Additionally, spikes had to be separated by a minimum time interval to ensure interspike intervals were larger than 1 ms. SU responses to all repetitions of the same stimulus were combined into one poststimulus time histogram (PSTH) with bins of 5 ms.

The raw signals from all LMA sites were also recorded and digitized (100-kHz sampling rate) for offline analysis to extract multiunit activity (MUA). LMA signals were first digitally bandpass filtered offline (0.3–4.5 kHz, three-pole Butterworth), then, extreme values (>2 SDs) were clipped, and finally, the short-term root-mean-squared (RMS) amplitude was calculated. To calculate the RMS, the signal was squared, lowpass filtered ( 200Hz, 2nd-order Butterworth), down-sampled to 500Hz, half-wave rectified, and square-rooted (Stark and Abeles, 2007). This process for extracting MUA was performed for each stimulus repetition and then the median MUA signal across all repetitions was computed to obtain a single response for each reverberant condition. The analyses in this paper are based on either the 241 available MU recordings or only the 103 MU recording sites from which an SU was also identified.

### Spectrogram reconstruction from neural responses to speech

#### Stimulus spectrogram

A spectrographic representation of the speech stimuli S(t,f) (Fig. 1*D*) was created using a gammatone filterbank, that was implemented with Slaney’s MATLAB auditory toolbox (Slaney, 1993); here, t=1,...,T specifies the time samples for a speech stimulus, and $f=f_{min},...,f_{max}$ denotes the center frequencies of the filterbank. We used a filterbank with 30 gammatone filters with center frequencies scaled logarithmically from $f_{min}=250Hz$ to $f_{max}=8kHz$ (Slaney, 1993). Following Patterson et al. (1992), the equivalent rectangular bandwidths (ERB) of the gammatone filters were set to estimates of human psychophysical bandwidths given by Glasberg and Moore (1990). After applying the gammatone filterbank, we calculated the log-amplitude Hilbert envelope in each frequency band (Theunissen et al., 2000) and the envelope signals were resampled to 200 Hz for a temporal resolution of 5 ms. Finally, the signal was clipped at an amplitude of −100 dB relative to the peak of each spectrogram, and bias was added to get positive spectrogram values (Fig. 1*D*).

We also experimented with spectrograms of higher frequency (up to 60 frequency bands) and higher temporal (1-ms time steps) resolutions. However, these higher resolutions did not qualitatively change the results; thus, 30 frequency bands and 5-ms time steps proved to be a good compromise between spectrogram resolution and computational burden.

#### Reconstructed spectrograms

We used the spectrogram reconstruction technique (Bialek et al., 1991; Stanley et al., 1999; Mesgarani et al., 2009) to quantify the amount of information about the speech utterances contained in the measured ensemble responses for both SUs and MUs. By comparing the reconstruction quality for the various reverberant conditions, we evaluated the effect of reverberation on the neural code.

**Figure 2.**
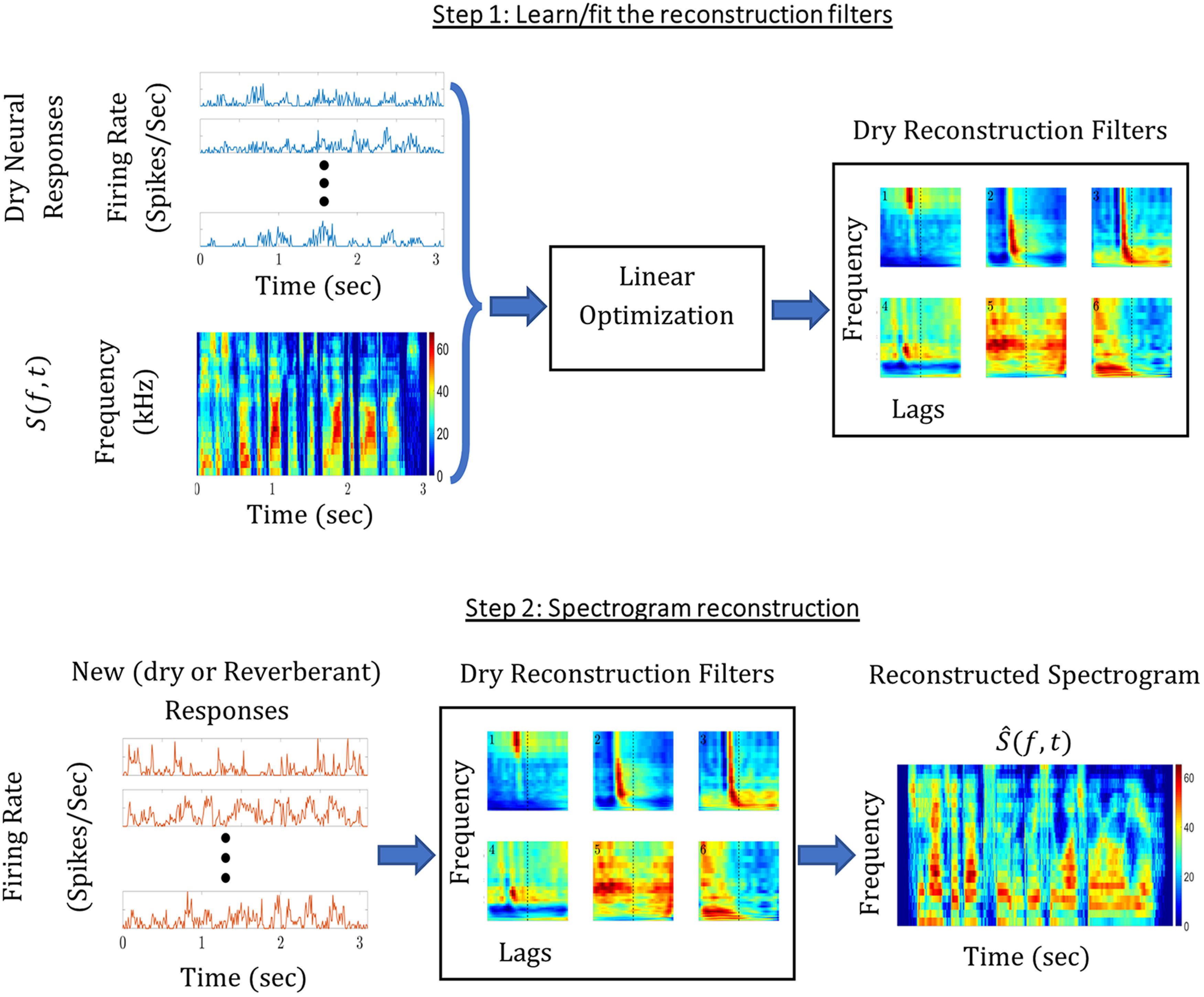
Linear spectrogram reconstruction. To quantify the amount of stimulus information available in neural responses, we used the optimal linear reconstruction technique applied to spectrograms. In Step 1, stimulus spectrograms in the dry condition and the corresponding measured responses of an ensemble of units (“dry responses”) are used to derive the optimal reconstruction filter (“dry filter”). The dry filter is optimal in that it minimizes the mean-square error between the stimulus spectrogram and a reconstructed spectrogram for the training data. In Step 2, a different set of neural responses are used with the dry filters to reconstruct the spectrogram for each reverberant condition. We use cross-validation between the two steps, such that the dry filter is derived from a subset of the data while the reconstruction accuracy is determined for the remaining subset not used for training. For each unit, the dry reconstruction filter is a two-dimensional matrix consisting of weights along frequency (*y*-axis) and lag (*x*-axis). We used noncausal reconstruction filters that can have nonzero weights for both positive and negative lags. The 30 frequency weights range from 250 Hz to 8 kHz on a log frequency scale, as for the stimulus spectrograms. Temporal weights range from −30 to +30 ms in 5-ms steps.

The reconstruction process (Fig. 2) is an optimal linear mapping, in the least square sense, between the measured neural response and the spectrographic representation of the stimulus, S(t,f). We separately analyzed two types of neural responses: poststimulus time histograms (PSTHs) with 5-ms resolution for SUs, and the median envelope response, sampled at the same 5-ms temporal resolution for MUs.

The reconstruction process consists of two steps: (1) fitting the optimal linear reconstruction filters g(t,n) for the set of n=1,...,N measured responses (SU or MU) to the dry speech stimulus, and (2) processing the measured responses in either dry or reverberant conditions through the reconstruction filter to yield the reconstructed spectrogram $\hat{S}(t,f)$ for the corresponding condition.

The optimal linear mapping in Step 1 can be calculated one frequency band at a time (Mesgarani et al., 2009). Denoting the reconstructed spectrogram for each frequency band f as ${\hat{S}}_{f}(t)$, we used linear regression to estimate the 30 linear filters $g_{f}(t,n)$ corresponding to the frequency bands of the input spectrogram f and the n recorded responses. In most cases (except for the data in Fig. 9), these filters were estimated from a training set of responses to dry utterances only. The reconstruction for each frequency (Fig. 2, Step 2) was performed by convolving the learned optimal linear filter with a test response set (i.e., PSTH for SU or median envelope response for MU), which could be from dry or reverberant conditions:

(1) $${\hat{S}}_{f}\left(t\right)=\underset{n}{\sum }g_{f}\left(t,n\right)*R\left(t,n\right)=\underset{n}{\sum }\underset{\tau }{\sum }g_{f}\left(\tau ,n\right)R\left(t-\tau ,n\right).$$

Here, the operator * is the linear convolution in the time domain, n is the index of unit responses (either SU or MU), and R(t,n) is a matrix consisting of the delayed responses of the nth neural unit to the test stimulus (Mesgarani et al., 2009).

Theoretically, the solution for the reconstruction filters $g_{f}(t,n)$ in Equation 1 is given in matrix form as $g_{f}=C_{RR}^{-1}C_{RS_{f}}$, where $C_{RR}=RR^{T}$ is the autocorrelation matrix of the neural response for the training set and $C_{RS_{f}}=RS_{f}^{T}$ is the cross-correlation matrix between the neural response and the stimulus spectrogram (Mesgarani et al., 2009). In practice, the matrix $C_{RR}$ is close to singular that is, its smallest singular values are just numerical noise. Simply inverting $C_{RR}$ will amplify these noisy singular values, hence obscuring the desired reconstruction features. To overcome this difficulty, we used ridge regression and calculated the pseudo-inverse matrix of $C_{RR}$. Specifically, we masked the smallest (noisy) singular values by adding a fixed small real scalar λ (Theunissen et al., 2001; David and Gallant, 2005). The optimal $\lambda_{opt}$ would minimize the mean-squared error between the stimulus spectrogram and the reconstructed spectrogram. To find $\lambda_{opt}$ we performed jackknife resampling using the 11 dry “training set” utterances and recalculated the inversion with 14 logarithmically scaled values for λ ranging from $10^{-5}\cdot s_{max}$ to $1.0\cdot s_{max}$, where $s_{max}$ is the maximum singular value of the matrix $C_{RR}$. This value of λ was fixed for all analyses.

This study aimed to evaluate the total amount of information available in neural responses for optimal linear reconstruction. To that end, the reconstruction filters $g_{f}$ were constructed to be noncausal in the sense that past and future samples were used to reconstruct $\hat{S}\left(t,f\right)$ at time t. Specifically, the filters $g_{f}$ extended ±30ms from time t=0 ms at a temporal resolution of 5ms (thus each filter was 13 samples long). We also experimented with longer lags ( ±50ms) and finer temporal resolutions (down to 1ms), but the results did not change qualitatively.

When estimating the reconstructed spectrograms $\hat{S}\left(t,f\right)$, we used leave-one-out cross-validation to avoid overfitting and reduce reconstruction variability (Theunissen et al., 2001; David and Gallant, 2005). This was done by estimating an optimal linear filter over 11 of the 12 utterances in the stimulus set for the dry condition (Step 1) and testing the reconstruction quality over the excluded utterance for all other conditions (dry and reverberant; Step 2). These steps were repeated for all 12 utterances, resulting in 12 scores of reconstruction quality. The median of these 12 scores was used to get an overall score for each DRR condition. We used the median rather than the mean because of its robustness to outliers. Reconstruction quality was quantified by the Pearson correlation coefficient (CC) between the stimulus and reconstructed spectrograms. For each utterance, the correlation coefficient was summed over all time samples and all frequency bands, and the results were averaged over the 12 utterances. We also compared results using the mean-squared error between original and reconstructed spectrograms (not shown) and the results were qualitatively the same.

### Data analysis

#### Determination of characteristic frequency from FRA

For single units in which we measured an FRA, we estimated the characteristic frequency (CF) as the frequency where activity can be detected at the lowest sound level. To determine the CF, we first interpolated the FRA heatmap grid by a factor of 10 along both axes for a finer resolution. Next, the unit’s background activity was removed by keeping the 5th to 10th percentile of the firing rates (for example, 95−100% for an excitatory unit and 0−5% for an inhibitory unit). Then the iso-rate contours of the interpolated map were identified (MATLAB image processing toolbox). Finally, the CF was set to the frequency of the lowest point along the longest contour. This algorithm worked quite well for most typical IC units but could give erroneous results for FRAs that were too noisy, or for units that had disjoint activity zones. In such cases, and if possible, we manually corrected the CF to the approximate frequency point as defined above.

#### Best correlated frequency and best stimulus envelope

**Figure 3.**
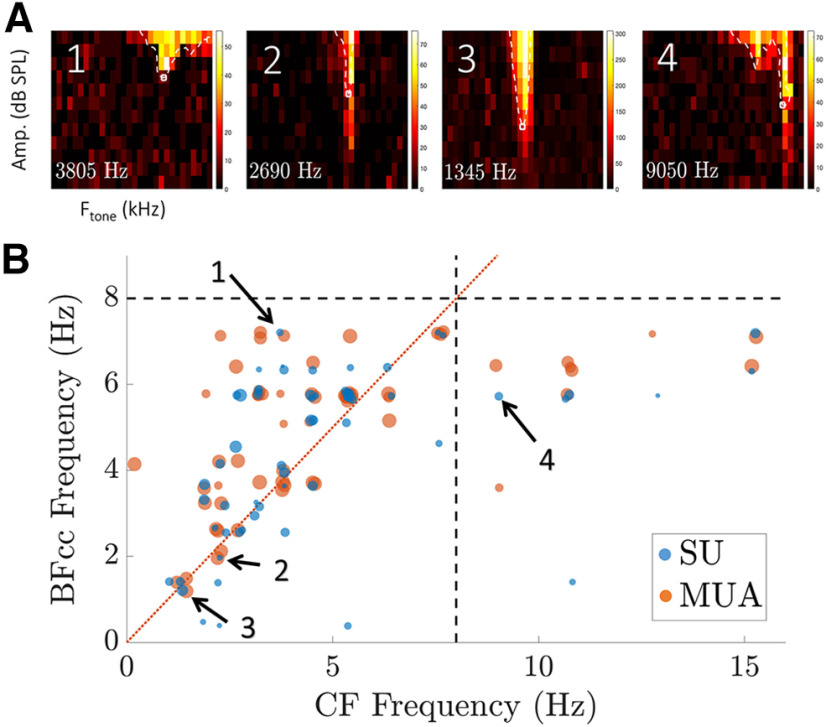
Comparing pure-tone characteristic frequency (CF) with best frequency of correlation coefficient (BF_CC_) for speech stimuli. ***A***, Frequency response area (FRA) of four neurons with CFs of 3805, 2690, 1345, and 9050 Hz. ***B***, Scatter plot of CF against BF_cc_, for the 65 units (SU or MU) in which an FRA was measured. Each dot corresponds to one measurement (SU or MU), and the symbol size is proportional to the CC between the best envelope and the measured response to speech. Across the 56 recording sites with CF < 8 kHz, there is a weak correlation between CF and BF_CC_ [SU: *R*^2^ = 0.65, *p* < 10^−4^ root-mean square error (RMSE) = 1.41; MU, *R*^2^ = 0.63, *p* < 10^−4^, RMSE = 1.27].

A simple way to compare a speech stimulus with the response of single or multiunits is by correlating the two signals. Because the spectrograms represent the speech envelopes in each of 30 frequency bands, and IC neurons are known to exhibit frequency selectivity, finding which of the 30 spectrogram envelopes shows the greatest correlation with the neural response provides a way to assess the unit’s frequency selectivity for speech stimuli. Therefore, for each unit in our sample (SU or MU) we computed the Pearson cross-correlation between the neural response and each of the speech spectrogram’s 30 band envelopes. The stimulus envelope yielding maximum correlation with the measured response was termed “the best envelope,” and the center frequency of the corresponding gammatone filter was defined as the best-correlated frequency (BF_cc_). BF_cc_ is a measure of the unit’s frequency selectivity for complex stimuli that can be compared with the CF, which is a measure of frequency selectivity for pure tone stimuli (Fig. 3*B*). The Pearson cross-correlation between the best envelope for the dry stimulus and the neural response (SU or MU) to each reverberant condition was one metric used to quantify the effect of reverberation on the encoding of speech at a given recording site.

#### Neural modulation depth and modulation gain

**Figure 4.**
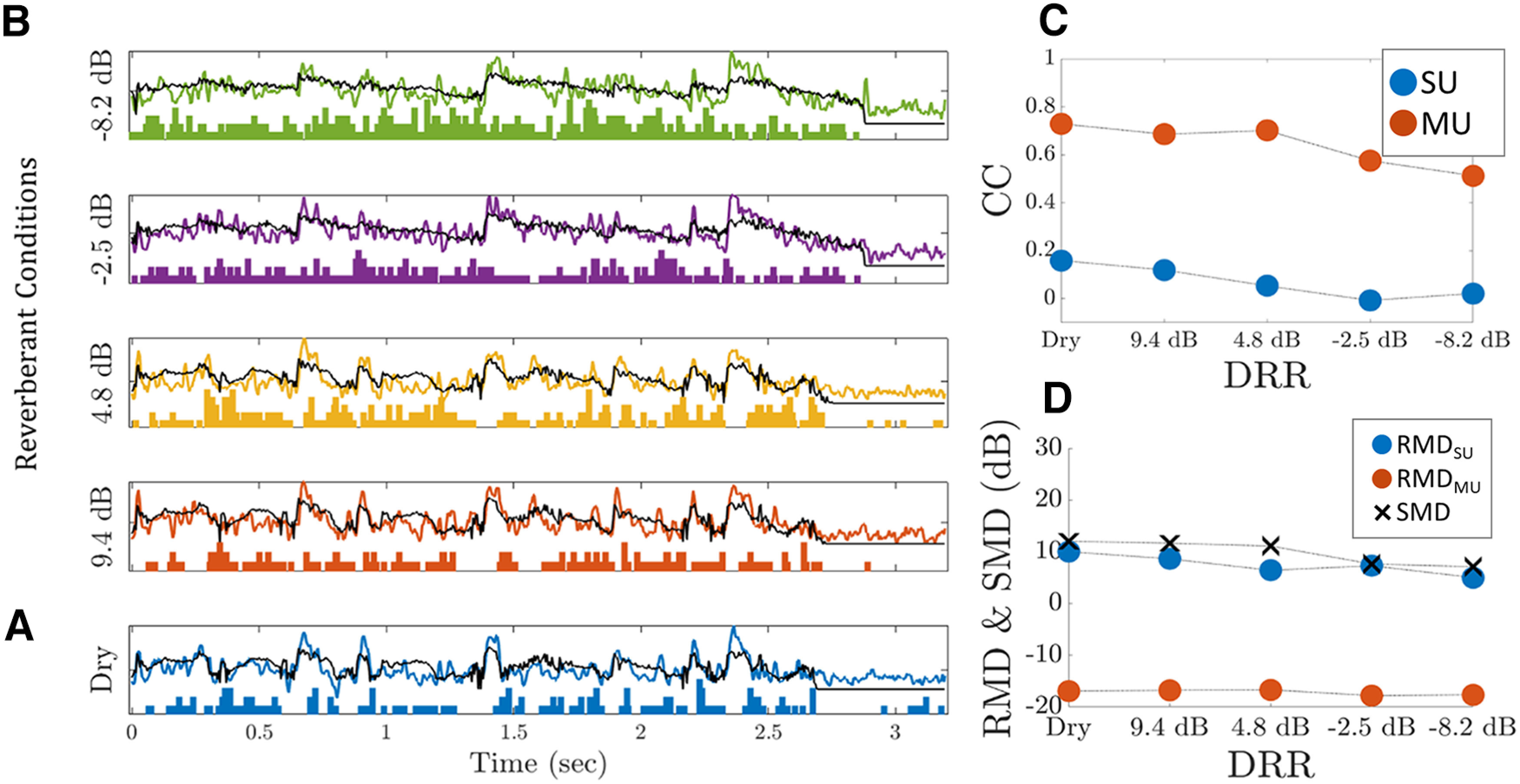
Response of a single unit (SU) and a multiunit (MU) from the same IC recording site to a speech utterance presented in various reverberant conditions. The utterance “Growing well-kept gardens is very time consuming” was pronounced by a female speaker. ***A***, Poststimulus time histogram (PSTH; 5-ms bin width, blue bars) of the SU and median MU response (continuous blue line) to the dry speech. The black line shows the best fitting stimulus envelope (the output of the gammatone filter centered at BF_CC_) for the dry condition. ***B***, Same as in *A* for each reverberant condition identified by the DRR on the left. All panels share the same time scale, but amplitudes were scaled to facilitate comparison. ***C***, Pearson correlation coefficient (CC) between the neural response and the envelope of the dry speech at the output of the gammatone filter centered at BF_CC_ as a function of DRR for both the SU and the MU. ***D***, Response modulation depth of the SU (RMD_SU_), the MU (RMD_MU_), and stimulus modulation depth (SMD) as a function of DRR.

Since reverberation is known to reduce perceptually important modulations in speech (Houtgast and Steeneken, 1973, 1985), we also quantified the amount of modulation in the neural response to characterize how reverberation affects the encoding of speech (Kuwada et al., 2014; Slama and Delgutte, 2015). Specifically, for each level of reverberation, we defined the response modulation depth (RMD) as $\sqrt{2}$ times the ratio of the SD (across time) of the neural response (PSTH for SU, median envelope for MU signal) to its mean value. The $\sqrt{2}$ factor is introduced so the value of RMD is consistent with the standard definition of modulation depth if the modulation is sinusoidal. The neural modulation gain MG was defined as the ratio of RMD to the modulation depth of the best stimulus envelope (SMD, the modulation depth of envelope at the output of the gammatone filter yielding the highest correlation with the neural response) for the same level of reverberation. In this paper, modulation depths (RMD and SMD) and modulation gains were usually expressed and plotted in dB (Fig. 4*D*).

#### Stimulus spectrograms correlation coefficients

We computed the correlation coefficients between dry and reverberant stimulus spectrograms (stimulus-CCs) to serve as a benchmark against which to assess the stimulus reconstruction quality in the various reverberant conditions. The stimulus spectrogram for each reverberant condition was vectorized and cross-correlated (Pearson correlation) with the vectorized dry stimulus spectrogram, each frequency band at a time. This resulted in five CC values, one for each reverberant condition, including the dry condition where CC equals 1. These values are a useful benchmark because the stimulus-CCs refer only to stimuli and do not involve neural measurements.

Because the CC is bounded between −1 and +1, its sampled distribution is biased, and this bias depends on the values of the correlations themselves. Thus, for the parameter testing results for all CCs in this work, we used Fisher’s z-transform to transform the CC distributions into variables with approximately Gaussian (normal) distributions with constant variances.

#### Time-dependent correlation coefficients

The time-dependent correlations (CC_t_) are a measure of the neural population response’s ability to encode the speech stimulus on an instant-by-instant basis. To assess how reconstruction quality varies throughout an utterance, we computed the CC_t_ between short temporal segments of the dry spectrogram $\left(S_{dry}\right)$, the most reverberant spectrogram $\left(S_{-8.2dB}\right)$, and the reconstructed spectrogram $\left({\hat{S}}_{-8.2dB}\right)$. Specifically, the time-dependent correlation coefficients were calculated for pairs of 5-ms bins, each containing 30 frequency bands, corresponding to consecutive time steps in our spectrographic representation. As with the long-term average CCs, all parameter testing on the CC_t_ was performed over their respective Fisher’s z-transform.

#### Identification of voiced and unvoiced segments

Dry speech segments were classified into voiced and unvoiced segments to compare spectrogram reconstruction quality for the two types of phones. The presence of voicing was identified using the probabilistic YIN (pYIN) algorithm in the Librosa Python toolbox (McFee et al., 2015). The pYIN algorithm is a probabilistic modification of the YIN algorithm (de Cheveigné and Kawahara, 2002) with improved performance; like YIN, it uses the autocorrelation function but adds another postprocessing phase in which the most probable candidate for a pitch value at each time segment is selected. For that process, the algorithm defines the most probable regions of voiced speech.

We applied the pYIN algorithm to our monophonic human voice recordings from the TIMIT corpus with a sampling rate of 16 kHz. For our purpose, the algorithm performed well with the default values from the Librosa library. The number of frequency bands used by the algorithm was set to 1024 and the hop length to 128 samples (8 ms).

#### Code accessibility

The code described in the paper is freely available online in the link https://github.com/odedbarz/RevPublish.

## Results

### Effects of reverberation on single-unit and multiunit responses to speech

We measured the responses to natural speech utterances of 103 single neurons and 241 multiunits from the inferior colliculus (IC) of three unanesthetized Dutch-belted rabbits. Note that the 103 single-unit recordings are from a subset the 241 multiunit recording sites. The stimulus set consisted of 12 speech utterances from the TIMIT corpus. Each stimulus was presented both without reverberation (dry condition) and with 4 degrees of reverberation, ranging from low (DRR=9.4dB) to high (DRR=−8.2dB).

Figure 4*A* shows the responses of an example single unit (SU; blue bars) and the multiunit (MU; blue line) from the same recording site to a dry speech utterance.

To estimate the frequencies to which the recording site is tuned to, we cross-correlated the MU response to dry speech with the speech envelopes in each of the 30 frequency bands comprising our spectrographic representation. The band envelope yielding the maximum correlation with the dry MU response (the “best envelope”) is shown by the black curve in Figure 4*A*. The best envelope follows the MU response reasonably well ( $r^{2}=0.73$, $p<10^{-4}$). This best envelope was obtained at the output of the filter with a center frequency of 7100 Hz, which we call the “best correlation frequency” (BF_cc_).

Figure 4*B* shows responses of the same SU and MU as in Figure 4*A* to the same utterance presented in four degrees of reverberation. The reverberant speech envelope at the output of the filter centered at BF_cc_ is also shown as black lines. Note that BF_cc_ is determined for the dry condition only and then the corresponding filter is used to generate speech envelopes in the different reverberation conditions.

The response to the dry stimulus in Figure 4*A* is more discrete and localized along the time axis relative to responses to the reverberant stimuli in Figure 4*B*. That is, as reverberation increases (DRR decreases), the neural responses (PSTHs for SU and envelope responses for MU) tend to be more blurred and elongated. This degradation is most severe for the offset responses, while onset responses are less affected. For example, the MU onset response (blue line) in the dashed dark rectangle in Figure 4*A* (1.325–1.5 s) closely follows the rise in the stimulus envelope (Fig. 4*A*, black line). The same holds for the MU responses during that time interval for the reverberant conditions (Fig. 4*B*), although the envelopes of the reverberant stimuli (Fig. 4*B*, black lines) do not show the same prominent onset. Overall, however, such robustness to reverberation is the exception, especially for high reverberation ( DRR<0dB). This is evident, for example, when comparing the reverberant MU response for DRR=−8.2dB (Fig. 4*B*, green line) and the reverberant stimulus envelope (Fig. 4*B*, black line) in that the MU response follows the stimulus envelope tightly.

We used two complementary metrics to quantify how reverberation alters the neural representation of speech by each unit. First, we calculated the Pearson cross-correlation (CC) between each reverberant neural response (SU or MU) and the best envelope of the dry stimulus. A constant CC across the different levels of reverberation would indicate that the reverberant neural responses remain similar to the dry stimulus envelope. The second metric used to quantify the effect of reverberation is the response modulation depth RMD which characterizes the fluctuations in the neural response relative to the mean response for each level of reverberation (see Materials and Methods). The two metrics are complementary in that the CC measures the shape similarity between the neural response and the dry stimulus envelope but is not sensitive to modulation depth, whereas RMD measures the amount of neural modulation without reference to a stimulus. The RMD was also compared with the amount of modulation in the stimulus envelope at the same level of reverberation to compute the neural modulation gain MG (see Materials and Methods) which quantifies how neural processing attenuates or amplifies the modulations present in the stimulus.

Figure 4*C* shows the envelope-to-neural CCs as a function of DRR for both the SU and the MU from the example recording site. For both unit types, the CC decreases with increasing reverberation, indicating a degraded neural representation of the dry stimulus envelope. At this recording site, the CC is much larger for the MU than for the SU. Figure 4*D* shows RMD as a function of DRR for the SU and the MU. Also shown is the modulation depth SMD in the best stimulus envelope for each reverberant condition. As expected, SMD decreases with increasing reverberation because of the effect of room acoustics. RMD for the SU also decreases somewhat, but not as much as the SMD, suggesting that, for this SU, the degradation in the envelope representation caused by reverberation is smaller for the neural response than for the stimulus. RMD for the multiunit was much smaller than RMD for the SU, even in the dry condition. These low RMD values arise because the modulations in the MU responses are superimposed on a large DC component (Fig. 4*A*,*B*). The initial step in the computation of the MU response is square-law rectification followed by lowpass filtering, which will transform broadband noise in the recording into a DC component. Since we do not have an independent method to estimate the amount of noise in the recording, we could not assess its contribution to the DC in the MU response and its effect on RMD. Because the observation of very low RMDs for MU responses was systematic across our sample of neurons, we only report RMDs for SU responses in the following text. Noise in the recording does not directly affect the SU response since the PSTH depends only on the timing of spikes and not their amplitudes. Noise also does not affect the CC between the MU response and the best stimulus envelope since the Pearson CC is insensitive to the addition of a DC component.

**Figure 5.**
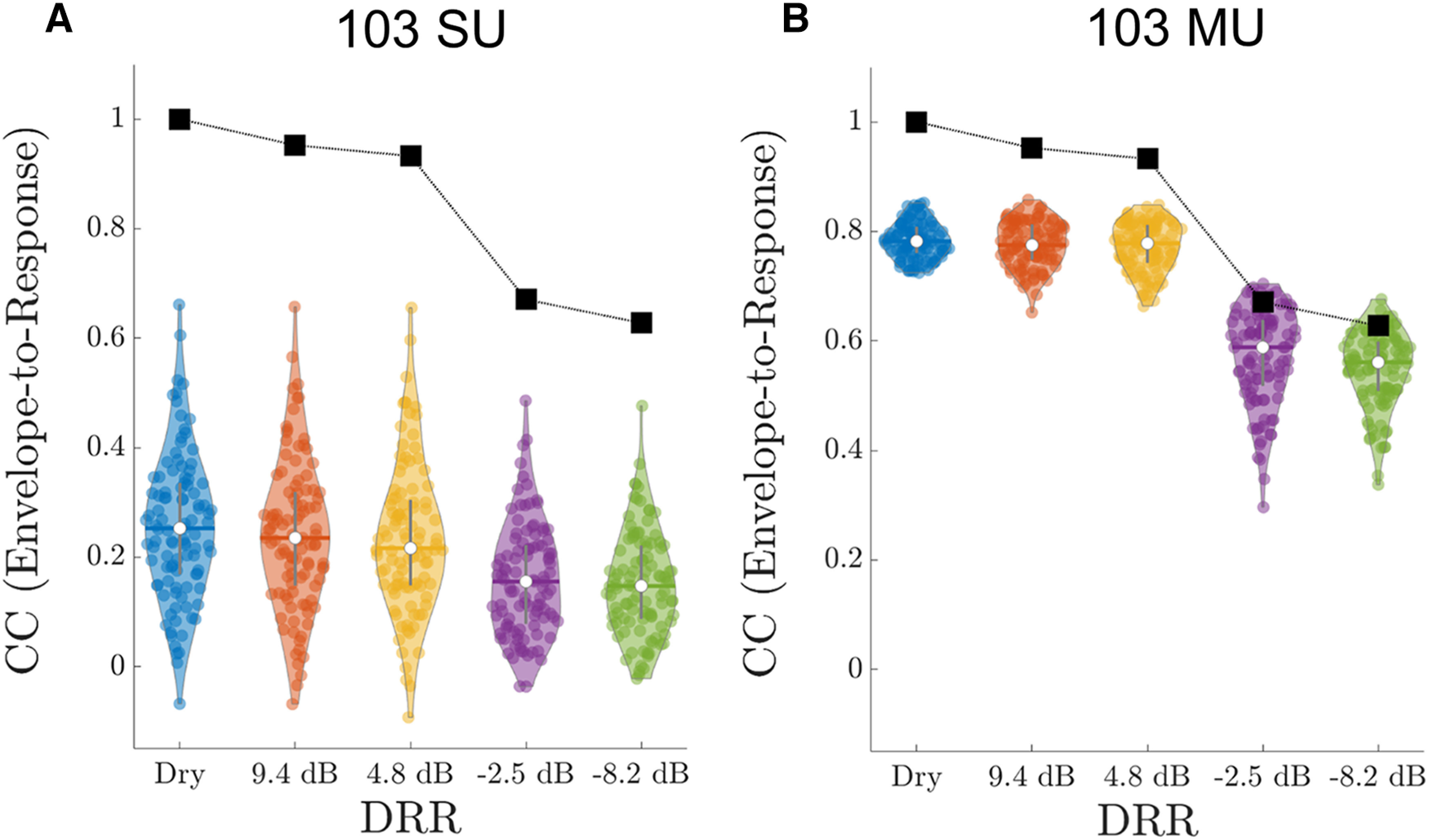
Effect of reverberation on responses of SUs (***A***) and MUs (***B***) to speech across the neural population. For each SU or MU (colored circle), we calculated the Pearson correlation coefficient (CC) between the neural response and the envelope of the dry speech at the output of the gammatone filter centered at BF_CC_. The white circles show the median CC across the population for each DRR. For both SUs and MUs, the median CCs decrease monotonically with increasing amount of reverberation (decreasing DRR), although the CCs for SUs are much lower and show greater variability than CCs for MUs. The black lines and squares show the stimulus CCs computed between the dry and reverberant stimulus spectrograms (i.e., not including neural processing).

The trends observed in the example in Figure 4 are representative of the neural population. Figure 5*A*,*B* shows the envelope-to-neural CCs as a function of DRR for our entire sample of SUs and MUs, respectively. The median CCs (white circles) decrease monotonically with increasing reverberation for both SUs and MUs, indicating an increasingly degraded neural representation of the dry stimulus. The mean envelope-to-neural correlations are higher for MUs (median: 0.782 in dry-condition and 0.561 for DRR=−8.2dB) than for SUs (median: 0.252 in dry condition and 0.147 for DRR=−8.2dB). Additionally, the variability in CCs across recording sites is smaller for MUs [interquartile range (IQR): 0.047 for dry condition and 0.087 for DRR=−8.2dB] compared with SUs (0.166 IQR for dry condition and 0.133 for DRR=−8.2dB).

As a reference, Figure 5 also shows the CC between dry and reverberant stimulus envelopes computed from the spectrograms (stimulus-CCs; black squares; see Materials and Methods). This metric quantifies the effect of reverberation on the stimulus spectrogram itself, without any neural processing. For all DRRs, the median envelope-to-neural CCs are smaller than the stimulus CCs, although the distribution of envelope-to-neural CCs for MUs overlaps somewhat with the stimulus CCs at the lowest DRRs.

We ran a two-way ANOVA on the envelope-to-neural CCs with the amount of reverberation (DRR) and unit type (SU vs MU) as fixed factors. A Fisher’s z-transform was preapplied to the CC values to approximate the normal distributions assumed by ANOVA. Both main effects were highly significant (DRR: *F*_(4,1020)_ = 274.8, *p* < 0.0001; Unit type: *F*_(1,2020)_ = 7555, *p* < 0.0001). There was also an interaction between DRR and unit type (*F*_(4,1020)_ = 102.8, *p* < 0.0001) reflecting the steeper decrease in CC with decreasing DRR for MUs compared with SUs. *Post hoc* paired comparisons with Tukey–Kramer corrections showed that each CC for DRR < 0 dB was significantly lower (*p* < 0.05) than any CC for DRR > 0 dB, but none of the other paired comparisons were significant. This split of the data into two groups (DRR < 0 dB vs DRR > 0 dB) held for both SUs and MUs.

**Figure 6.**
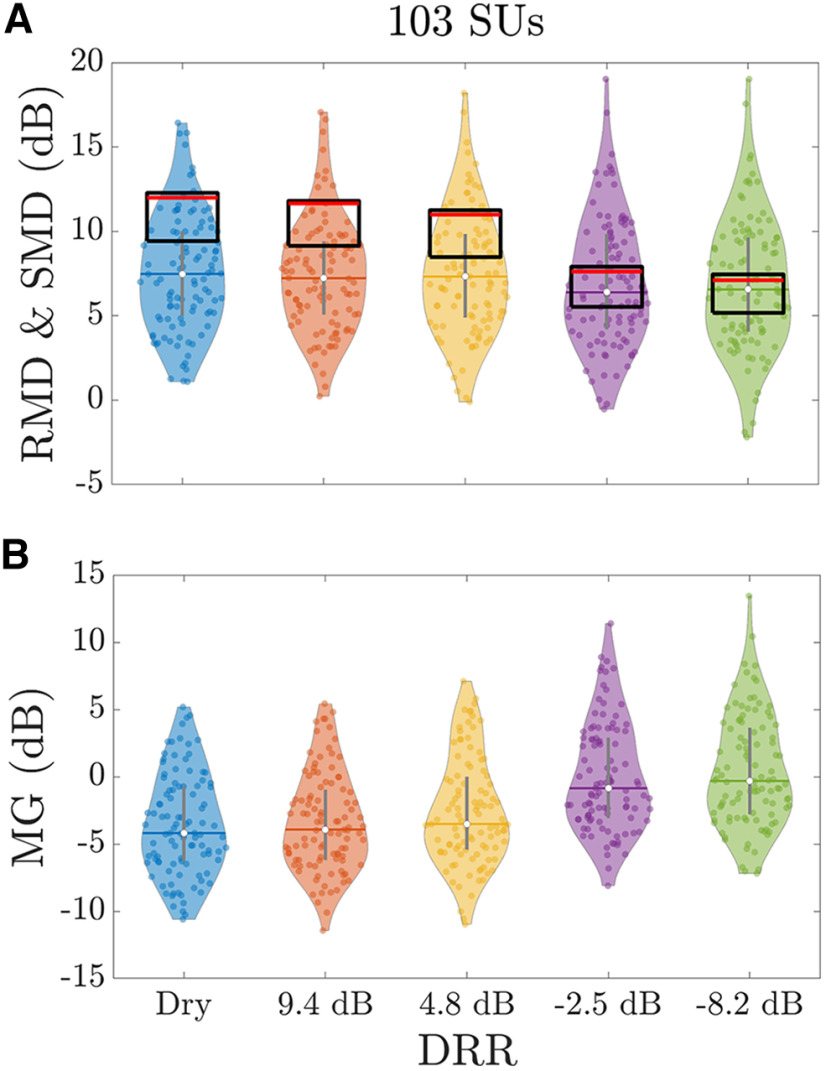
Reverberation affects the temporal coding of amplitude modulation in IC single units. ***A***, Response modulation depth (RMD; colored circles) and stimulus modulation depth (SMD; black rectangles) for the sample of 103 single-unit responses. For the SMD, the black rectangles show the 25th and 75th percentiles, and the red horizontal bars inside each of the black rectangles are the median SMDs across frequency channels for each DRR. Despite a slight trend for the median RMD to decrease with increasing reverberation, the effect was not statistically significant (Kruskal–Wallis test: *p* = 0.398, χ^2^ = 4.05, df = 4) because of the large variability in the data. However, the median SMDs clearly decreased with increasing amount of reverberation and approached the median RMDs for negative DRRs. ***B***, The neural modulation gain (MG), in dB, is the ratio of the RMD to the SMD for each unit. The median MG tended to increase with increasing reverberation (Kruskal–Wallis test: *p* < 10^−4^, χ^2^ = 80.1, df = 4); this observation is consistent with earlier findings using sinusoidally amplitude modulated (SAM) noise stimuli (Kuwada et al., 2014; Slama and Delgutte, 2015).

Figure 6*A* shows the RMD as a function of DRR for the 103 SUs in our sample. Also shown are the median and IQR stimulus modulation depths (SMDs) across the different gammatone frequency channels. The median RMD shows a slight monotonically decreasing trend with increasing amount of reverberation, but the maximum amount of change did not exceed 0.93 dB and was small compared with across-neuron variability in RMD. The maximum change in median RMD was also smaller than the 4.9 dB drop in median SMD from dry to the most reverberant condition. As a result, the median neural modulation gain MG (the log difference between RMD and SMD; Fig. 6*B*) increased by ∼4 dB with increasing reverberation. A decrease in RMD coupled with an increase in neural modulation gain has also been observed in studies of the effect of reverberation on the temporal coding of sinusoidal AM by IC neurons (Kuwada et al., 2014; Slama and Delgutte, 2015).

To verify these trends, we ran Kruskal–Wallis tests on both the RMD and the MG with DRR as a factor. There was no significant effect of DRR on the median RMD (χ^2^(4) = 4.05, *p* = 0.399). However, the effect of DRR on modulation gain was significant (χ^2^(4) = 80.13, *p* < 0.0001). *Post hoc* paired comparisons with Tukey–Kramer corrections showed that all the median modulation gains for negative DRRs (−2.5 and −8.2 dB) were significantly higher (*p* < 0.0001) than all the modulation gains for positive DRRs (Dry, +9.4 and +4.8 dB). No other paired comparisons were significant. Thus, the dichotomy between negative and positive DRRs observed for the stimulus-to-neural CC also holds for the neural modulation gain.

The analyses using stimulus-to-neural CCs and RMD/MG give complementary pictures of the effect of reverberation on the neural coding of speech by individual IC recording sites (SU or MU). The CCs between the neural response and the dry speech envelope decreased steeply with increasing reverberation and were higher for MU responses than for SU responses. The latter finding is not unexpected considering that MU signals are an average of the responses of many units that were recorded simultaneously near the recording site, while the PSTH of an SU represents the time-average spike count from just one neuron. On the other hand, reverberation had only minimal effects on the modulation depth of SU responses and these effects were smaller than the decrease in stimulus modulation depth, resulting in an increase in neural modulation gain with increasing reverberation, consistent with earlier results using sinusoidal AM stimuli (Kuwada et al., 2014; Slama and Delgutte, 2015). This increase in neural modulation gain may be indicative of the robustness of the temporal coding of AM by IC neurons to reverberation (see Discussion).

### Comparison of frequency tuning for speech versus pure-tone stimulation

The above results are based on comparing the neural response with the speech envelope at the output of the gammatone filter that yielded the maximum stimulus-response correlation for dry speech. The center frequency of this filter (BF_CC_) can be interpreted as a measure of the frequency selectivity of the recording site for complex sounds that can be compared with the CF derived from the FRA, which characterizes frequency selectivity for pure tones. Figure 3*B* is a scatter plot of BF_CC_ against CF for the 65 recording sites in which the FRA was measured. The BF_CC_ are limited to 8 kHz, which is the bandwidth of the TIMIT corpus and the highest frequency of our spectrographic representation, while pure tone CFs can extend up to the 18 kHz frequency limit of our acoustic system, hence no direct relationship between BF_CC_ and CF is expected for CFs above 8 kHz. Limiting the analysis to the 56 recording sites with CFs ≤ 8 Hz, there was a significant correlation between BF_CC_ and CF for both SUs (*R*^2^ = 0.65, *p* < 10^−4^) and MUs (*R*^2^ = 0.63, *p* < 10^−4^). There was, however, a great deal of scatter in the data. This scatter may reflect differences in neural frequency selectivity for speech versus pure tones because of nonlinear processing in the cochlea and brainstem. It may also be because of the highly co-modulated nature of speech, such that speech envelopes from different frequency bands can be similar even if the two bands are widely separated in frequency (Singh and Theunissen, 2003; Elhilali and Shamma, 2008; Atiani et al., 2009; Viswanathan et al., 2022).

### Linear spectrogram reconstructions resemble spectrograms of dry speech in mild reverberation

To quantify the amount of speech information available in the responses of the neural population, we used linear stimulus reconstruction techniques (Bialek et al., 1991; Mesgarani et al., 2009) to reconstruct the stimulus spectrogram from ensemble neural activity. Optimal mean-squared error reconstruction filters (“dry-filters”) were derived from a training set of responses to the dry stimulus and for various ensembles of units (Materials and Methods). We then applied these dry filters to the responses to both dry and reverberant speech (testing set) to obtain reconstructed spectrograms that were compared with the spectrogram of the original dry speech. Our premise was that, if there is a dereverberation process in the IC, we expect reconstructed reverberant spectrograms to be more similar to the dry stimulus spectrograms than to the reverberant speech spectrograms, as found by Mesgarani et al. (2014) in ferret auditory cortex.

**Figure 7.**
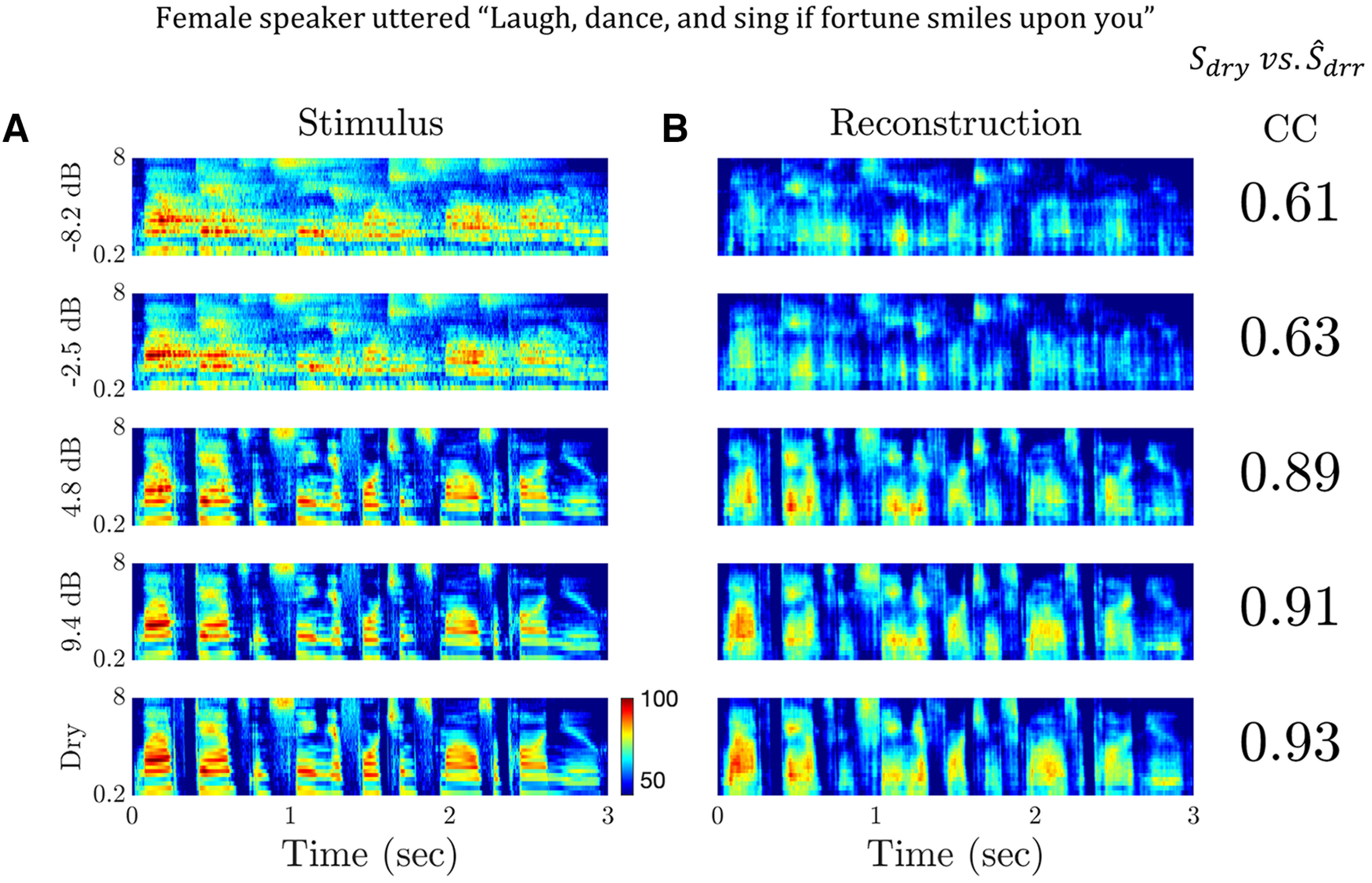
Linear spectrogram reconstructions for dry and reverberant speech. ***A***, Stimulus spectrograms of an utterance in dry and four reverberant conditions. ***B***, Corresponding linear spectrogram reconstructions based on the responses of 241 multiunits. Increasing reverberation degrades the reconstruction quality, as measured by the Pearson cross-correlation (CC) between the reconstruction ( ${\hat{S}}_{DRR}$) and the dry stimulus spectrogram ( $S_{dry}$). However, reconstruction quality remains high (CC > 0.89) so long as the DRR is > 0 dB. Severe degradation only occurs for negative DRRs.

Figure 7*A* shows the stimulus spectrograms of an utterance produced by a female speaker for both the dry condition ( $S_{dry}$) and in four degrees of reverberation ranging from mild to severe. Figure 7*B* shows the reconstructed spectrograms ${\hat{S}}_{drr}$ for each reverberant condition. For these reconstructions, we used all available MU recordings (241 MU responses). On the right of each reconstruction is the CC score, which is the Pearson correlation between the stimulus spectrogram $S_{dry}$ and the reconstructed spectrogram ${\hat{S}}_{drr}$ where the correlation is computed over both time and frequency. The reconstruction quality is very high for the dry condition (CC = 0.93), confirming that ensemble multiunit activity in the IC contains sufficient information to recover the speech features represented in the spectrogram.

As noted above, increasing reverberation degrades the spectro-temporal modulations, offsets, and onsets, in the stimuli (Fig. 7*A*). These detrimental effects also affect the reconstruction quality (Fig. 7*B*), as measured by the Pearson correlation coefficients. For the utterance of Figure 7, reconstruction quality remains good (CC=0.89) in mild reverberation (DRR=4.8 dB) but drops rapidly (CC=0.61) when reverberation further increases (DRR=−2.5 dB) with little further degradation for the lowest DRR ( −8.4 dB).

### Spectrogram reconstruction quality degrades with increasing reverberation and shows no evidence of a neural dereverberation mechanism

**Figure 8.**
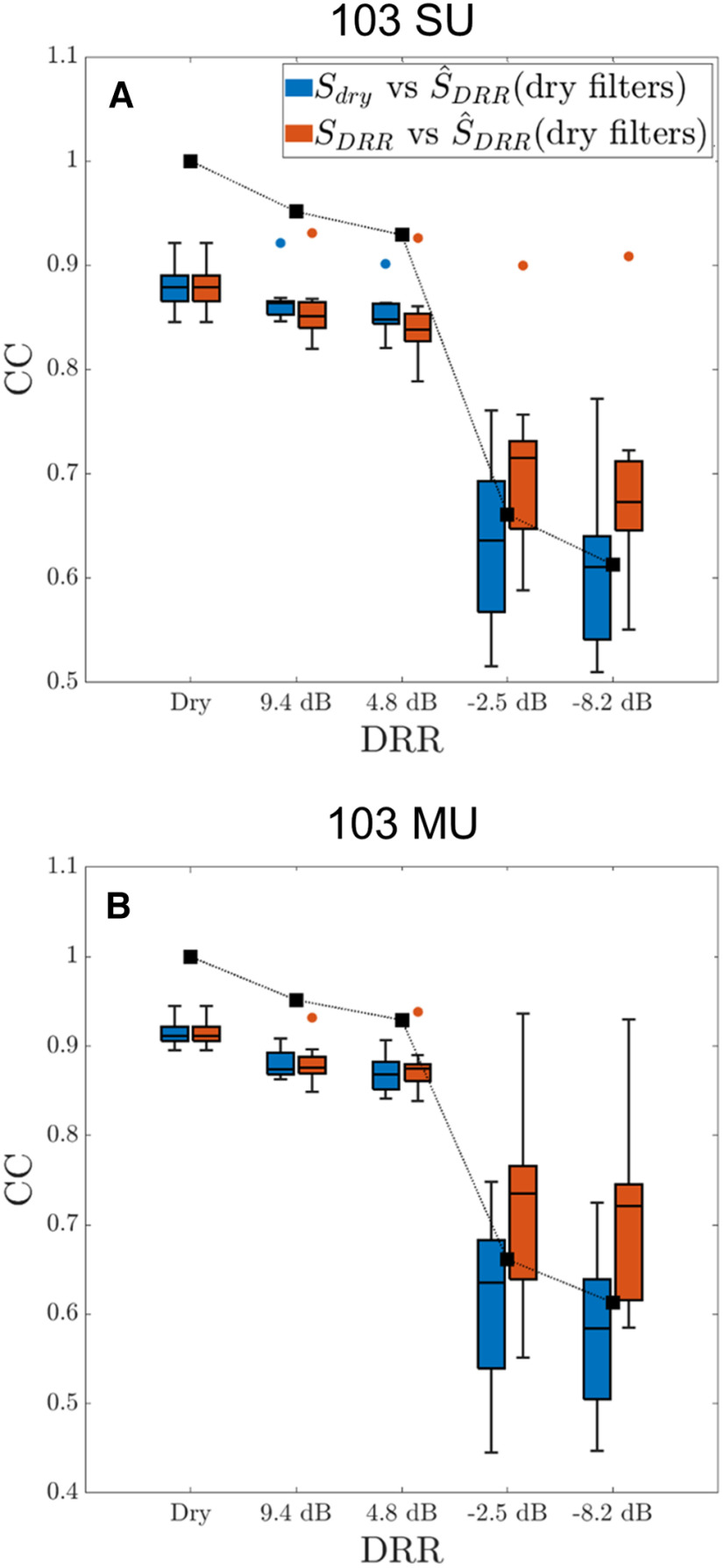
Spectrogram reconstruction quality from ensemble neural responses degrades with increasing amount of reverberation and shows no evidence for a dereverberation process for both SUs (***A***) and MUs (***B***). For each DRR, boxplots show the distributions of CC scores for reconstruction quality across the 12 TIMIT utterances used as stimuli. Two CC scores are shown for each DRR. The $S_{dry}$-vs- ${\hat{S}}_{DRR}$ score (blue bars) is the CC between the dry spectrogram $S_{dry}$ and the reconstructed spectrogram ${\hat{S}}_{DRR}$. The $S_{DRR}$-vs- ${\hat{S}}_{DRR}$ score (red bars) is the CC between a reverberant stimulus spectrogram $\left(S_{DRR}\right)$ and the reconstructed spectrogram ${\hat{S}}_{DRR}$ for the same DRR. Both scores were computed with reconstruction models that were trained with dry stimuli (dry-filter models). Black squares show the stimulus-only CCs between dry and reverberant spectrograms. Each boxplot shows the median, the interquartile range (IQR), and the nonoutlier minimum and maximum. Outliers (circles) are defined as having values >1.5 IQR above the upper quartile.

Figure 8 shows the distributions (in the form of boxplots) of CC scores for spectrogram reconstruction quality across all 12 stimulus utterances as a function of DRR for reconstructions from both SU (Fig. 8*A*) and MU ensemble responses (Fig. 8*B*). Two measures of reconstruction quality (CC scores) are shown for each DRR. The $S_{dry}$-vs- ${\hat{S}}_{DRR}$ scores (blue bars) are the CCs between the reconstructed spectrogram ${\hat{S}}_{DRR}$ and the *dry* stimulus spectrogram $S_{dry}$. The $S_{DRR}$-vs- ${\hat{S}}_{DRR}$ scores (red bars) are the CCs between the reconstructed spectrogram ${\hat{S}}_{DRR}$ and the stimulus spectrogram for the same DRR $\left(S_{DRR}\right)$. Both CCs scores were computed with models that were trained with dry stimuli (dry-filters). To ensure a meaningful comparison between SUs and MUs, the MU reconstructions were based on the subset of 103 measurements taken from the same recording sites from which the SU responses were measured.

For both SUs and MUs, both the $S_{dry}$-vs- ${\hat{S}}_{DRR}$ score and the $S_{DRR}$-vs- ${\hat{S}}_{DRR}$ score degrade with increasing amount of reverberation, with a large drop in reconstruction quality when the DRR falls below 0 dB. The boxplots show considerable variability in CC scores among the 12 utterances and several data points for SUs were found to be outliers (circles) according to boxplot conventions (see caption). These outliers all came from the utterance “Growing well-kept gardens is very time-consuming” pronounced by a male speaker. We have no explanation for why the spectrogram of this particular utterance was reconstructed especially accurately from ensemble neural activity.

**Figure 9.**
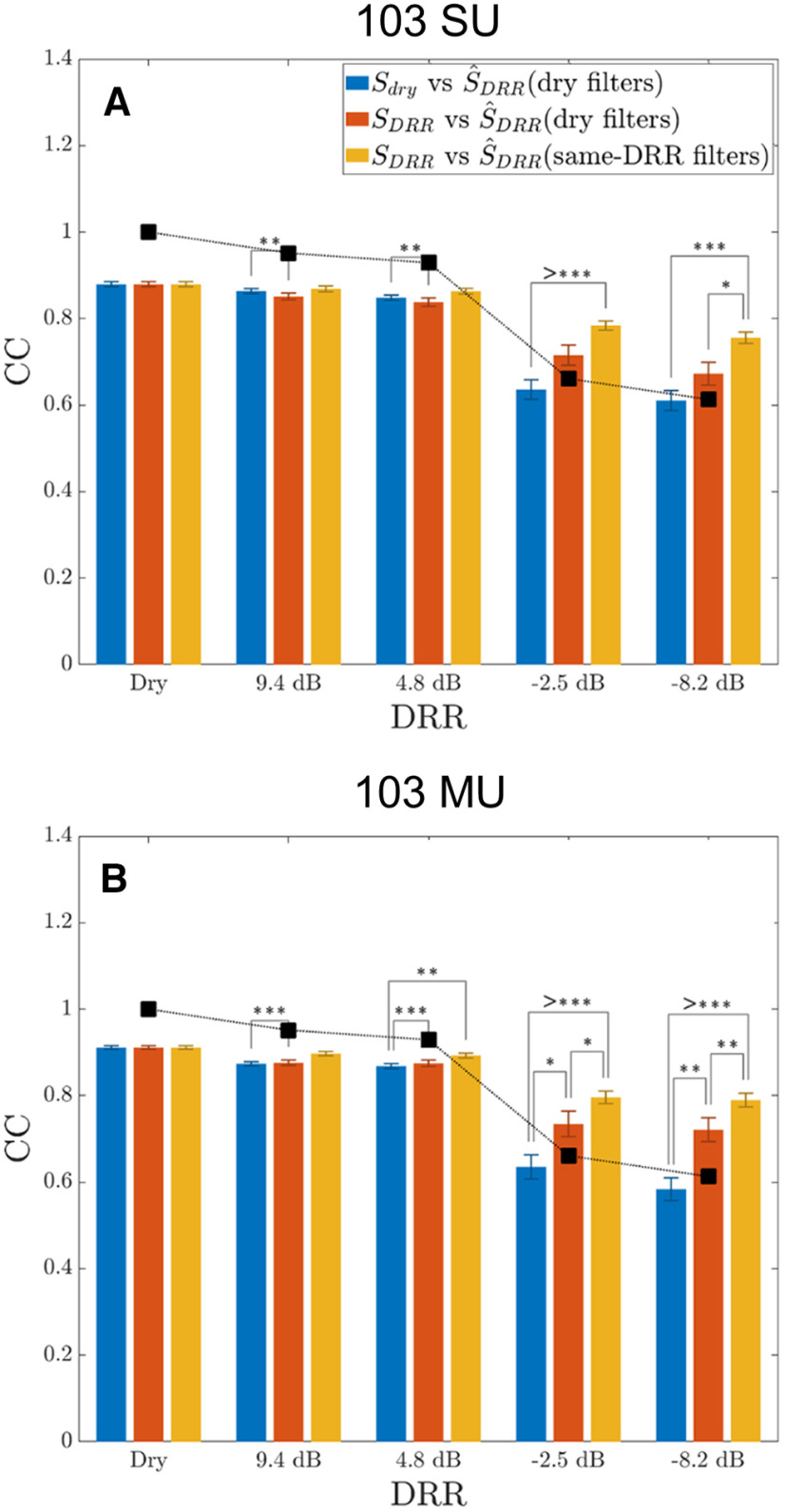
Three factors contribute to the observed degradation in reconstruction quality with increasing reverberation for both SUs (***A***) and MUs (***B***). Three reconstruction methods differing in the contribution of each factor are compared as a function of the amount of reverberation (DRR). The $S_{\mathrm{DRR}}$-vs- ${\hat{S}}_{DRR}$ (same-DRR filters) correlation coefficients (yellow bars) represent the degradation because of envelope tracking errors, which is the inability of the linear reconstruction model to perfectly track the stimulus envelope when the model is trained and tested with stimuli with the same degree of reverberation. The $S_{\mathrm{DRR}}$-vs- ${\hat{S}}_{DRR}$ (dry filters) scores (red bars) include the additional degradation because of model generalization failure, which refers to the model’s inability to capture new reconstructions when trained with dry stimuli and tested with reverberant stimuli. Finally, the $S_{dry}$-vs- ${\hat{S}}_{DRR}$ (dry filters) CC scores (blue bars) include the additional effects of distortion compensation failure, which is the inability to compensate for the distortion of the original speech envelope introduced by reverberation. Black squares show the stimulus-only CCs; *p*-values for *post hoc* paired comparisons between reconstruction methods based on two-way repeated measures ANOVA test are shown (see text).

To assess the possibility of a dereverberation process in the ensemble response of the IC neurons, we compared the CC scores between $S_{dry}$-vs- ${\hat{S}}_{DRR}$ and $S_{DRR}$-vs- ${\hat{S}}_{DRR}$ (Fig. 9, blue and red bars). The underlying assumption is that, if there exists a dereverberation process, the optimal reconstruction should be more similar to the dry stimulus spectrogram than to the reverberant stimulus spectrogram, as found by Mesgarani et al. (2014) in ferret auditory cortex. For both SUs and MUs, the CC distributions for the two reconstruction methods were largely overlapping in mild reverberation (DRRs of +9.4 and +4.8 dB), meaning the dry and reverberant spectrograms were equally well reconstructed. In more severe reverberation (DRRs of −2.5 and −8.2 dB), however, the CC scores for $S_{DRR}$-vs- ${\hat{S}}_{DRR}$ (red) tended to be higher than the CCs for $S_{dry}$-vs- ${\hat{S}}_{DRR}$ (blue), which is the opposite of the predicted result if there were a neural dereverberation process. This trend was confirmed by a detailed statistical analysis presented in the next section (Fig. 9). The steep degradation in reconstruction quality (CC scores for $S_{dry}$-vs- ${\hat{S}}_{DRR}$) between DRR>0 and DRR<0 was qualitatively similar to the corresponding drop in stimulus-only CCs (black squares), suggesting the decrease in the reconstruction quality was primarily driven by the effect of reverberation on the stimulus itself. Overall, these results provide no evidence for a dereverberation process for highly reverberant ( DRR<0) stimuli in the rabbit IC, at least not using the present analysis and quantification metrics.

### Factors affecting the degradation of spectrogram reconstruction quality with increasing reverberation

While Figure 8 demonstrates a degradation in spectrogram reconstruction quality from ensemble neural response with increasing reverberation, this degradation can result from at least three main factors: envelope tracking errors, model generalization failure, and distortion compensation failure. Envelope tracking errors refer to the inability of the linear reconstruction model trained on a given reverberant condition to reconstruct new unseen spectrograms in the same reverberant condition. Model generalization failure refers to the additional degradation in reconstruction quality when the model trained with responses to dry stimuli is tested against reverberant spectrograms. Distortion compensation failure refers to the inability to compensate for the distortion of the original speech envelope introduced by reverberation. The stimulus-only CCs (Fig. 9, black squares) demonstrate this third type of degradation; each CC is computed between the dry stimulus envelope and a reverberant stimulus envelope for the same utterances and for the same speaker.

To determine the relative importance of these three factors in the observed degradation in reconstruction quality with increasing reverberation, Figure 9 compares CC scores for reconstruction quality for a hierarchy of three reconstruction methods differing in the degree to which each factor contributes to the degradation. Two of these methods (Figs. 8, 9, blue and red bars) trained the linear reconstruction model using responses to dry speech only. The additional reconstruction method (Fig. 9, yellow bars) differs from the other two in that the linear model was trained on stimuli with the same degree of reverberation rather than from dry stimuli. These $S_{DRR}$-vs- ${\hat{S}}_{DRR}$ (same-DRR filters) scores are the CC between stimulus $\left(S_{DRR}\right)$ and reconstructed spectrograms ${\hat{S}}_{DRR}$ at the same-DRR so that the CC scores only represent the degradation because of envelope tracking errors. In contrast, for the $S_{DRR}$-vs- ${\hat{S}}_{DRR}$ (dry-filters) method (Figs. 8, 9, red bars), the model was trained with dry stimuli but the reconstructed spectrogram from reverberant stimuli ${\hat{S}}_{DRR}$ is compared with the stimulus spectrogram for the same DRR $S_{DRR}$. Thus, the scores reflect the combined effects of model generalization failure and envelope tracking errors. Comparing the CC scores for the $S_{DRR}$-vs- ${\hat{S}}_{DRR}$ (same-DRR filters) method (yellow bars) against the scores for the $S_{DRR}$-vs- ${\hat{S}}_{DRR}$ (dry-filters) method (red bars) isolates the additional contribution of model generalization failure. Finally, for the $S_{dry}$-vs- ${\hat{S}}_{DRR}$ (dry-filters) reconstructions (blue bars), the model was trained with dry stimuli and the spectrograms reconstructed from responses to reverberant stimuli were compared with dry stimulus spectrograms. Thus, these scores combine the detrimental effects of all three factors. The contribution of distortion compensation failure to the degradation can be isolated by comparing CC scores for the $S_{DRR}$-vs- ${\hat{S}}_{DRR}$ (dry-filters) method (red bars) versus the $S_{dry}$-vs- ${\hat{S}}_{DRR}$ (dry-filters) reconstructions (blue bars).

To quantitatively compare reconstruction quality for the three reconstruction methods, we ran two-way, repeated measures (RM) ANOVAs on the Fisher-transformed CC scores, with DRR and reconstruction method as within-subject factors. The “subjects” were the 12 TIMIT utterances used as stimuli. Data for the dry condition were excluded because they are identical for the three reconstruction methods. Separate analyses were run for the SU and MU scores. For MUs, there were highly significant effects of both DRR (*F*_(3,33)_ = 138.5, *p* < 0.0001) and the reconstruction method (*F*_(2,22)_ = 37.1, *p* < 0.0001), as well as a significant interaction between the two (*F*_(6,66)_ = 44.8, *p* < 0.0001). *Post hoc* paired comparisons with Bonferroni corrections showed that the $S_{DRR}$-vs- ${\hat{S}}_{DRR}$ (same-DRR filters) scores (yellow bars) were significantly higher (*p* < 0.05 or lower) than the $S_{DRR}$-vs- ${\hat{S}}_{DRR}$ (dry-filters) scores (red bars) for all four DRRs, suggesting that model generalization failure contributes to the degradation observed with the $S_{DRR}$-vs- ${\hat{S}}_{DRR}$ (dry filters) method. In addition, the $S_{DRR}$-vs- ${\hat{S}}_{DRR}$ (dry-filters) scores (red bars) were significantly higher than the $S_{dry}$-vs- ${\hat{S}}_{DRR}$ (dry-filters) scores (blue bars) for all negative DRRs (−2.5 and –8.2 dB), suggesting that distortion compensation failure also contributes to the degradation in reconstruction quality in severe reverberation with the $S_{DRR}$-vs- ${\hat{S}}_{DRR}$ (dry-filters) method. Thus, all three factors contribute to the degradation in reconstruction quality based on MU responses, with envelope tracking error being the most important factor. Importantly, distortion compensation failure also contributes in severe reverberation, providing specific evidence against the existence of a neural dereverberation process in the IC.

RM ANOVA results for SUs showed similar trends as for MUs but were less robust. Again the main effects of DRR (*F*_(3,33)_ = 239, *p* < 0.0001) and reconstruction method (*F*_(2,22)_ = 17.0, *p* = 0.0004) were highly significant, as was the interaction between the two factors (*F*_(6,66)_ = 14.9, *p* = 0.0006). The difference between $S_{DRR}$-vs- ${\hat{S}}_{DRR}$ (same-DRR filters) scores (yellow bars) and $S_{DRR}$-vs- ${\hat{S}}_{DRR}$ (dry-filters) scores (red bars) was significant for +4.8 and +9.4 dB DRRs, thereby showing a different pattern compared with MUs, where the difference was larger for lower DRRs. The $S_{DRR}$-vs- ${\hat{S}}_{DRR}$ (dry-filters) scores (red bars) differed significantly from the $S_{dry}$-vs- ${\hat{S}}_{DRR}$ (dry-filters) scores (blue bars) only for −8.2 dB DRR, although the comparison grazed significance (*p* = 0.058) for −2.5 dB DRR. The less robust results obtained with SUs compared with MUs may be because of the large variability in reconstruction quality across stimulus utterances, including outliers for SUs. Nevertheless, all three factors contribute to the degradation in reconstruction quality for SUs as well as MUs.

#### Good quality spectrogram reconstruction can be achieved from responses of 25–50 units

**Figure 10.**
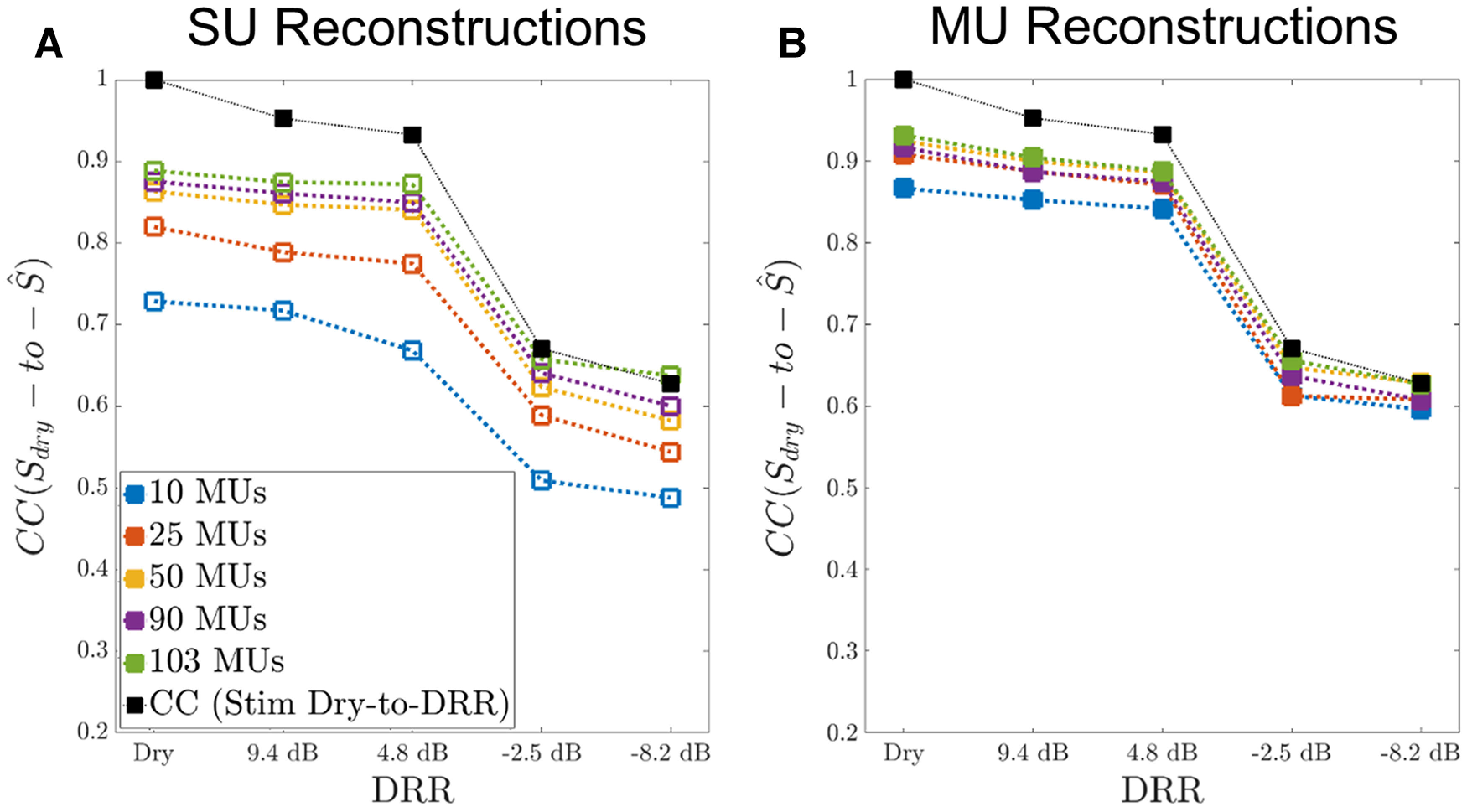
Quality of spectrogram reconstruction from ensemble responses of both SUs (***A***) and MUs (***B***) improves with increasing ensemble size and is better for MUs than for SUs for small ensemble sizes and modest reverberation. For each amount of reverberation, reconstruction quality was quantified by the Pearson correlation coefficient (CC) between the dry stimulus spectrogram and the corresponding spectrogram reconstruction. This was done for neural ensembles of various sizes. Twenty-five MU measurements sufficed to reach an asymptote in reconstruction quality for all DRRs, that is, adding more units to the ensemble did not improve CCs substantially. With ensemble of size ≥50, reconstruction quality was high (CC > 0.8) for dry and mild reverberation conditions (DRR > 0 dB) but deteriorated markedly in severe reverberation (DRR < 0 dB). Reconstruction quality was higher when based on MUs than when based on SUs, especially for small ensemble sizes and low reverberation. Black squares show stimulus CCs between the dry and reverberant stimulus spectrograms (i.e., not including neural responses). This benchmark was reached in severe reverberation for reconstructions based on MUs.

So far, spectrogram reconstruction quality has only been analyzed for neural ensembles containing large numbers of units (241 MUs in Fig. 7; 103 SUs, and 103 MUs from the same sites in Figs. 8, 9). Next, we analyze how reconstruction quality varies with ensemble size. Figure 10 shows the median CC scores for the $S_{dry}$-vs- ${\hat{S}}_{DRR}$ (dry-filters) reconstruction method as a function of the number of units (both SUs and MUs) used for the reconstruction. To facilitate meaningful comparison, the subsets of MUs used for this analysis were always selected from the same recording sites as the corresponding subset of SUs. The CC scores for the largest ensemble of 103 units (Fig. 10, green squares) are the same as in Figure 9, blue bars. For the smaller subsets (i.e., 10, 25, 50, and 90 units) we performed 11 iterations; in each iteration, units were selected randomly and without repetition from the sample of 103 units. To obtain the final CC score shown in Figure 10, the median score was first computed for each utterance over these 11 iterations, and then the median of these medians was computed over the 12 stimulus utterances.

Figure 10 shows that reconstruction quality degrades systematically with decreasing ensemble size and increasing reverberation (decreasing DRR) and is clearly better for MUs than for SUs for small ensemble sizes. For example, the CC scores for 10 MUs are higher than the corresponding SUs scores for dry and mild reverberation conditions ( $CC_{SU,10}=0.728$ vs $CC_{MU,10}=0.866$ for the dry condition; $CC_{SU,10}=0.668$ vs $CC_{MU,10}=0.841$ for DRR=4.8dB). As the ensemble size used for reconstruction increases, the reconstruction quality improves markedly for SUs while the MU CC scores are already saturated for an ensemble size of 25 units so that the SU scores approach the MU scores. This effect is especially evident for DRR >0  dB. In high reverberation ( DRR=−8.2  dB). the CC scores for reconstructions from ensembles of 50 and 90 units are approximately the same for SUs and MUs ( $CC_{SU,50}=0.582$ vs $CC_{MU,50}=0.628$; $CC_{SU,90}=0.6$ vs, $CC_{MU,90}=0.607$). Thus, the benefit of having additional information in the MU responses compared with SU responses, which is apparent for small ensemble sizes and mild reverberation tends to vanish for large ensemble sizes and strong reverberation.

We ran a three-way ANOVA on the Fisher-transformed CC scores, with ensemble size, amount of reverberation (DRR), and unit type (SU vs MU) as factors. The CC scores for each of the 11 iterations and each of the 12 utterances were treated as a separate data points, giving a total of 6000 data points. Data for the ensemble size of 103 were excluded because only one iteration was available. The main effects of each factor were all highly significant (ensemble size: *F*_(4,5966)_ = 1125, *p* < 0.0001; DRR: *F*_(4,5966)_ = 8796, *p* < 0.0001; unit type: *F*_(2,5966)_ = 3129, *p* < 0.0001), as were all the two-way interactions between pairs of factors. The interaction between ensemble size and unit type (*F*_(4,5966)_ = 186, *p* < 0.0001) reflects the steeper dependence of CC scores on ensemble size for SUs compared with MUs. The interaction between ensemble size and DRR (*F*_(16,5966)_ = 39.6, *p* < 0.0001) reflects the smaller effect of ensemble size on CC scores at lower DRRs (strong reverberation) compared with higher DRRs. The interaction between DRR and unit type (*F*_(2,5966)_ = 279, *p* < 0.0001) reflects the different shapes of the dependence of CC scores on DRR for SUs versus MUs, with the former being more graded compared with the steep down step around 0 dB DRR for MUs.

### Reconstruction quality fluctuates over time in parallel with degradation in stimulus spectrogram

**Figure 11.**
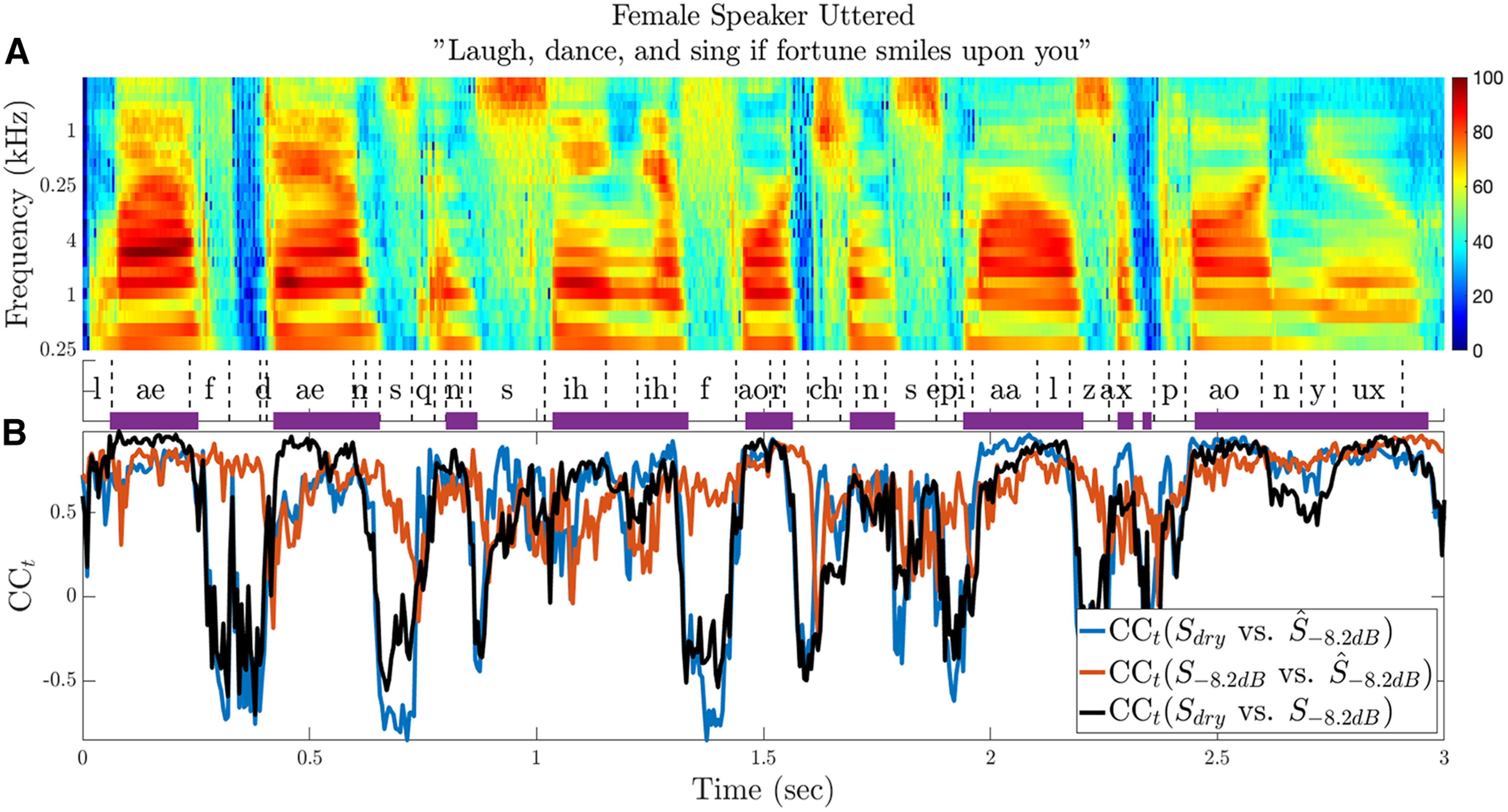
Temporal variations in spectrogram reconstruction quality over the course of an utterance. ***A***, Dry stimulus spectrogram of the utterance “Laugh, dance and sing if fortune smiles on you” pronounced by a female speaker. Selected phones are labeled below the spectrogram (not all phonemes are shown to avoid clutter). Purple horizontal lines show voiced segments identified using the probabilistic YIN (pYIN) algorithm. ***B***, Time-dependent correlations (CC_t_) were calculated between pairs of spectrograms using 5-ms time steps and over the whole frequency range (30 frequency bands). When assessed against the dry speech spectrogram, the quality of reconstruction derived from responses to reverberant speech fluctuates over time (blue curve). These fluctuations in reconstruction quality closely parallel the short-term cross-correlation between the dry and reverberant speech spectrograms (black curve), suggesting they are largely stimulus-driven. Fluctuations in reconstruction quality are less pronounced when assessed against the reverberant speech spectrogram (red curve).

So far, we have evaluated spectrogram reconstruction quality using the CC score, an overall metric that is averaged over the entire duration of each utterance. It is also of interest to examine how reconstruction quality varies over the course of an utterance and whether these variations relate to the phonetic structure. Figure 11*A* shows the spectrogram of the dry utterance “Laugh, dance and sing if fortune smiles on you” pronounced by a female speaker. Phonetic labels from the TIMIT corpus metadata (Garofolo et al., 1993) are shown under the spectrogram (Fig. 11*A*). The purple bars along the time axis indicate voiced segments, which were identified with the probabilistic YIN (pYIN) algorithm (Mauch and Dixon, 2014). Figure 11*B* shows time-dependent correlation coefficients (CC_t_) between the spectrograms for various stimulus conditions and the reconstructions based the 103 MU measurements taken from the same recording sites from which the SU responses were measured. The CC_t_ were calculated between two spectrograms for each time step (5-ms bins) and across all 30 frequency bands. The figure shows both the CC_t_ between the dry stimulus spectrogram and the highly reverberant reconstruction ( $S_{dry}\;vs\;{\hat{S}}_{-8.2dB}$; blue line) and the CC_t_ between the highly reverberant stimulus spectrogram and its reconstruction ( $S_{-8.2dB}\;vs\;{\hat{S}}_{-8.2dB}$; red line). For reference, the black line shows the stimulus-only CC_t_; that is, the time-dependent similarity between the dry spectrogram and the spectrogram of the highly reverberant stimulus $\left(S_{dry}\;vs\;S_{-8.2dB}\right)$.

**Figure 12.**
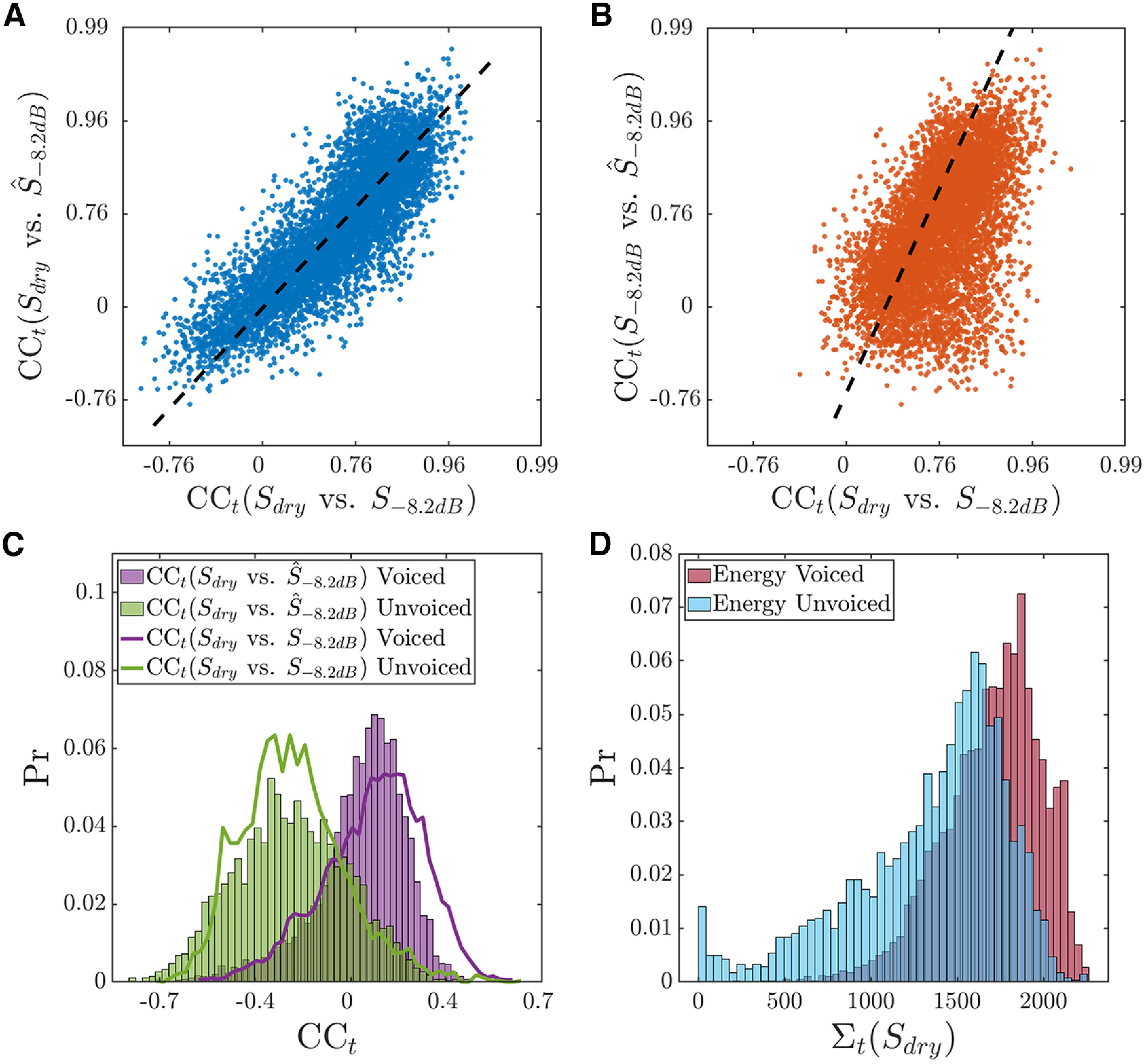
***A***, ***B***, Scatter plots of short-term cross-correlation (CC_t_) between pairs of spectrograms (time steps is 5 ms as in Fig. 11*B*). ***A***, Reconstruction scores CC_t_ between dry and reconstructed spectrograms (*y*-axis) are comparable (Pearson correlation test, $r_{t}=0.836$, $p<10^{-4}$, orthogonal regression slope: 1.08) to CC_t_ between the dry and reverberant speech spectrograms (*x*-axis). ***B***, Reconstruction scores of CC_t_ between highly reverberant spectrograms (DRR = −8.2 dB) and the corresponding reconstructed spectrograms show less resemblance (Pearson correlation test, $r_{t}=0.501$, $p<10^{-4}$, orthogonal regression slope: 2.19). ***C***, Reconstruction quality was estimated separately for voiced and unvoiced segments. Histograms show the distribution of Fisher-transformed CC_t_ between the dry stimulus spectrograms and the spectrogram reconstructed in strong reverberation (DRR = −8.2 dB) for voiced (purple bars) and unvoiced (green bars) segments. For comparison, the distribution of the CC_t_ between the dry stimulus spectrogram and the reverberant stimulus spectrogram (DRR = −8.2 dB) are shown for voiced (purple line) and unvoiced (green line) segments. Reconstruction quality was consistently high for voiced speech but varied widely for unvoiced speech. In addition, the reconstruction quality (colored bars) parallels the distributions of correlations between dry and reverberant speech spectrograms (colored lines). ***D***, Histogram of the distributions of stimulus energy for voiced and unvoiced segments computed from the dry spectrogram (silent segments were omitted). The energy distributions clearly overlap between voiced and unvoiced segments, in contrast to the more separated distribution of CC_t_ in ***C***. This suggests that differences in stimulus energy cannot entirely explain the greater reconstruction quality observed for voiced segments compared with unvoiced segments.

The CC_t_ between the dry speech spectrogram and the reverberant reconstruction ( $S_{dry}\;vs\;{\hat{S}}_{-8.2dB}$;Fig. 11*B*, blue line) closely parallels the CC_t_ between dry and reverberant speech spectrograms ( $S_{dry}\;vs\;S_{-8.2dB}$, black line), whereas the CC_t_ between the reverberant speech and its reconstruction ( $S_{-8.2dB}\;vs\;{\hat{S}}_{-8.2dB}$; red line) deviates more from the other two traces. To quantify this observation across the entire data set, Figure 12*A* shows a scatterplot for the instantaneous CC_t_ for $\left(S_{dry}\;vs\;{\hat{S}}_{-8.2dB}\right)$ against the stimulus-only CC_t_ $\left(S_{dry}\;vs\;S_{-8.2dB}\right)$ for all 12 utterances. The two variables are strongly correlated (Fisher-transformed CC_t_, *r*_t_ = 0.836, *p* < 10^−4^, orthogonal regression slope: 1.08). A similar scatter plot of CC_t_ for $S_{-8.2dB}\;vs\;{\hat{S}}_{-8.2dB}$ against CC_t_ for $S_{dry}\;vs\;S_{-8.2dB}$ (Fig. 12*B*) reveals a more modest, but still significant correlation (*r*_t_ = 0.501, *p* ≤ 10^−4^, orthogonal regression slope: 2.19). These results suggest that the temporal fluctuations in the quality of reconstruction of the dry stimulus from responses to the highly reverberant spectrogram are mostly driven by fluctuations in the similarity between the dry and reverberant stimulus spectrograms.

Figure 11*B* further shows that the reconstruction quality as measured by CC_t_ for $S_{dry}\;vs\;{\hat{S}}_{-8.2dB}$ is higher on average for voiced segments than for unvoiced segments. A similar observation holds for the stimulus only CC_t_ (black trace). In other words, voiced speech and its neural representation are, on average, more robust to the effect of reverberation than unvoiced speech. To quantify this observation over all 12 utterances, Figure 12*C* shows the distributions of the Fisher-transformed $CC_{t}\left(S_{dry}\;vs\;S_{-8.2dB}\right)$ for voiced and unvoiced stimulus segments (purple and green lines, respectively). The stimulus-only CC_t_ are, on average, higher for voiced segments (purple line) than for unvoiced segments (green line; Mann–Whitney *U* test, $z=51.6,p<10^{-4}$). Similarly, the CC_t_ between the dry stimulus spectrogram $S_{dry}$ and the reverberant reconstruction ${\hat{S}}_{-8.2dB}$ are significantly higher for voiced segments than for unvoiced segments (Fig. 12*C*, purple and green bars, respectively, Mann–Whitney *U* test, $z=47.2,p<10^{-4}$).

The greater robustness to reverberation of voiced sounds over unvoiced sounds may simply result from the greater amplitude of voiced sounds on average. If a high-amplitude voiced sound is preceded by a weak unvoiced sound [e.g., in a consonant-vowel (CV) syllable], the reverberant tails from the preceding unvoiced sound will have minimum influence on the spectro-temporal features of the stronger voiced sound. The opposite pattern is predicted if a low-amplitude unvoiced sound is preceded by a high amplitude voiced sound (e.g., in a CVC syllable). Figure 12*D* shows that, while voiced segments have higher amplitude than unvoiced segments on average, as expected, the overlap between the two energy distributions is greater than the overlap between the CC_t_ distributions in Figure 12*C*. This was confirmed by receiver operation characteristic (ROC) analysis to compare the performance of an ideal observer in discriminating voiced from unvoiced sounds based on the energy distributions (Fig. 12*D*) or on the CC_t_ distributions (Fig. 12*C*). The areas under the ROC curve were 0.701 and 0.763 for energy and CC_t_, respectively, suggesting that the greater robustness to reverberation of voiced sounds compared with unvoiced sounds cannot be entirely explained by amplitude differences.

## Discussion

We used natural speech utterances to test whether neural coding in the inferior colliculus is robust to reverberation. We recorded single-unit (SU) and multiunit (MU) activity from the IC of unanesthetized Dutch-belted rabbits in response to speech utterances under dry and various reverberant conditions. Realistic reverberation was introduced with binaural room impulse responses simulated by the room-image method (Allen and Berkley, 1979; Shinn-Cunningham et al., 2001). By comparing the temporal patterns of neural responses with the best-fitting speech envelope at the output of a filter bank approximating cochlear processing, we showed that the ability of individual SUs and MUs to track the dry stimulus envelope degraded with an increasing amount of reverberation, with an especially steep decrease for DRRs near 0 dB. On the other hand, reverberation produced only minimal reductions in the modulation of single-unit neural responses and these reductions were smaller than the reduction in stimulus envelope modulations at the same level of reverberation. To quantify the speech information available in population neural activity, we used optimal linear mappings to reconstruct stimulus spectrograms from ensemble neural responses. High-quality spectrogram reconstruction could be achieved from responses of ensembles of 50 SUs or 25 MUs to dry speech and mildly reverberant speech. However, reconstruction quality degraded with increasing reverberation, and the degradation was more severe when reconstruction quality was assessed against the dry speech spectrogram than when assessed against the speech spectrogram at the same level of reverberation. The dependence of both measures of reconstruction quality on the amount of reverberation roughly paralleled the similarity between dry and reverberant stimulus spectrograms, suggesting it was largely shaped by the properties of the stimulus.

### Effect of reverberation at individual recording sites

For single-site recordings from both SUs and MUs, we found that the median Pearson cross-correlation between neural responses and the best stimulus envelope for dry speech decreased monotonically with increasing reverberation, with the decrease being particularly steep for DRRs near 0 dB (Fig. 5). This finding indicates that increasing reverberation degrades the neural representation of the dry stimulus, although it also suggests a degree of robustness to mild-to-moderate reverberation. Robustness is also supported by the data on neural modulation depth, which showed only a slight decrease with increasing reverberation (Fig. 6*A*). Because the decrease in neural modulation depth was smaller than the corresponding decrease in stimulus modulation depth at the same level of reverberation, the neural modulation gain increased in stronger reverberation, indicating neural amplification of the small modulations associated with reverberant stimuli (Fig. 6*B*).

A degradation in the temporal coding of AM with increasing reverberation coupled with an increase in neural modulation gain has been observed in previous studies of the coding of SAM noise stimuli by IC neurons (Kuwada et al., 2012, 2014; Slama and Delgutte, 2015). For example, Kuwada et al. (2014) reported an increase in neural modulation gain of up to 10 dB as the distance between the sound source and the receiver increased in a virtual room. Slama and Delgutte (2015) found that the neural modulation gain was larger in reverberant conditions (with DRRs of 0 and –6 dB) than for anechoic stimuli in 86% of IC neurons. They related this increase in gain to the compressive shapes of modulation input-output functions (MIOFs), such that a given increment in stimulus modulation depth causes a larger increase in neural modulation depth when imposed on a small baseline modulation than on a large baseline modulation. Because the modulations present in the source signal get attenuated by room acoustics before they reach the ear, stimulus modulation depths are, on average, smaller in reverberant environments, thereby resulting in an increase in neural gain via MIOF compression. Compressive MIOFs are widely observed in IC neurons (Krishna and Semple, 2000; Nelson and Carney, 2007; Slama and Delgutte, 2015) as well as more peripheral neurons including those of the auditory nerve (Joris and Yin, 1992) and cochlear nucleus (Rhode and Greenberg, 1994; Sayles et al., 2013). The present results suggest that the relationship between compressive MIOFs and increased neural modulation gain in reverberation that was demonstrated for SAM noise may also apply to more spectro-temporally complex stimuli such as speech. The degree to which MIOF compression in auditory neurons depends on the modulation waveform and spectrum is unknown.

Because perceptual modulation detection thresholds for reverberant AM stimuli have been reported to be lower than predictions from room acoustics (BRIRs; Zahorik et al., 2011, 2012), it is tempting to interpret the increase in neural modulation gain with increasing reverberation as a neural compensation mechanism for the acoustic effects of reverberation. However, because the increase in gain occurs for both monaural and binaural stimulation (Kuwada et al., 2014) and the neural amplification of small modulations would be beneficial not only in reverberation but also in other conditions that attenuate modulations such as additive background noise, the increase in neural gain may represent a general mechanism for enhancing the temporal coding of small modulations rather than a specific reverberation compensation mechanism. This view is consistent with human psychophysical reports that modulation depth discrimination thresholds increase with increasing baseline modulation depth, which would be expected from compressive MIOFs (Ozimek and Sek, 1987; Wakefield and Viemeister, 1990; Ewert and Dau, 2004).

In addition to the increase in neural modulation gain with increasing reverberation, Slama and Delgutte (2015) observed a distinct form of reverberation compensation in that about one-third of IC neurons showed a “reverberant advantage,” whereby the neural modulation depth was larger for reverberant stimuli than for “anechoic” stimuli matched for modulation depth at the input to the ears. They hypothesized that this “reverberant advantage” was because of synergistic interaction between IC neuron’s sensitivity to AM and their sensitivity to fluctuations in interaural coherence (IAC; Joris, 2006). In reverberation, the fluctuating amplitude of AM sounds results in variations in the energy ratio of direct to reflected sounds reaching the ears, causing the IAC to fluctuate periodically. Recent work by Shaheen et al. (2022) provides physiological evidence for this “dynamic IAC” hypothesis. It is unclear whether this form of reverberation compensation operated with our speech stimuli as it does with SAM noise stimuli. Since the speech stimuli were presented binaurally using realistic BRIRs, the envelope modulations in the source signal likely resulted in concomitant IAC fluctuations on which the putative compensation mechanism operates. However, we did not perform the necessary manipulations of binaural stimulus properties to directly test this compensation mechanism.

### Single-unit and multiunit responses

We found that the MU responses to speech better represent the stimulus than the SU responses. This suggests that the responses of neighboring neurons (neurons in the vicinity of the recording site) in the IC have a correlated response which averages out SU noise or the inability of single units to fully follow a stimulus with their response. This interpretation is consistent with reports of similarities in the response properties of neighboring IC units (Seshagiri and Delgutte, 2007; Chen et al., 2012; Schnupp et al., 2015).

### A steep increase in the effects of reverberation for DRRs near 0 dB

Several features of our data showed a steep degradation in the neural coding of reverberant speech for DRRs near 0 dB, specifically when the DRR fell from +4.8 to –2.5 dB, contrasting with smaller effects for both lower and higher DRRs. This was the case for the median correlation between neural responses and the best stimulus envelope (Fig. 5) as well as for measures of spectrogram reconstruction quality from ensemble neural responses (Figs. 8, 10). Several factors likely contributed to this inflection point in neural coding. A steep decrease near 0 dB DRR also occurs in the correlation between dry and reverberant speech spectrograms (Fig. 8, black lines) suggesting the inflection already occurs in the stimulus. The two most reverberant conditions (–2.5 and –8.2 dB DRR) were generated with highly reflecting walls (20% absorption) and had much longer reverberation times (RT_60_ = 2.03 s) than conditions with positive DRRs (RT_60_ = 0.32 s). Thus, the steep degradation in neural coding near 0 dB may reflect a change in reverberation time rather than a change in DRR per se. However, early reflections are known to affect speech intelligibility in rooms (Bradley et al., 2003; Arweiler and Buchholz, 2011), so it is unlikely that the accuracy of neural coding is entirely determined by RT_60_, which does not depend on early reflections.

Our finding of a steep degradation in the neural coding of speech for DRRs near 0 dB is consistent with a human psychophysical report (Larsen et al., 2008) that perceptual sensitivity to changes in DRR [as measured by just-noticeable differences (JNDs)] is better for reference DRRs near 0 dB compared with both lower and higher reference DRRs. However, the task performed by Larsen and colleagues’ subjects was to judge the “degree of reverberance” of noise stimuli and did not require the processing of speech. Larsen et al. (2008) further show that several acoustic characteristics of their stimuli (such as IAC, spectral variance, and spectral center of gravity) have a sigmoid dependence on DRR with an inflection point near 0 dB, providing a possible basis for the enhanced perceptual and neural sensitivity in this range of DRRs.

### Neural population encoding of speech stimuli in reverberation

To quantify the population encoding of speech under the various reverberation conditions, we used an optimal linear reconstruction technique (Bialek et al., 1991; Stanley et al., 1999; Mesgarani et al., 2009) to reconstruct the stimulus spectrograms from an ensemble of SU or MU recordings. We quantitatively compared reconstructed spectrograms with dry and same-reverberation spectrograms and showed that reconstructed spectrograms for same-reverberant conditions resembled reverberant stimulus spectrograms more than dry stimulus spectrograms.

In a similar study, Mesgarani et al. (2014) measured responses of single units in the primary auditory cortex (A1) of awake ferrets to both speech stimuli and conspecific vocalizations presented either in reverberation or with additive noise. Stimulus spectrograms reconstructed from the recorded ensemble responses were found to be more similar to the clean (dry) stimulus spectrograms than to the noisy or reverberant spectrograms. The authors concluded that A1 neurons in the ferret were robust and maintained the same statistical distribution of response properties for both clean and distorted speech, including reverberant speech. These results from the ferret auditory cortex clearly differ from the present results from the rabbit IC.

In addition to the differences in species and recording sites, the Mesgarani et al. (2014) study differed from ours in how reverberation was simulated. To simulate reverberation, Mesgarani et al. (2014) convolved their source signals with random Gaussian noise with an exponentially decaying envelope (time constant 300 ms, which corresponds to RT_60_ = 2.07 s). This model for reverberation is oversimplified and does not include important characteristics such as the direct sound and early reflections. In particular, it does not allow computation of a DRR. For comparison, we implemented the Mesgarani et al. (2014) RIR and convolved it with our dry speech. The reverberant speech spectrograms created in this way most resembled our highly reverberant spectrograms (DRR < 0).

Additionally, the stimuli in the Mesgarani et al. (2014) study were presented monaurally and thus did not include the binaural characteristics of reverberant stimuli such as decorrelation. In contrast, we used the room-image algorithm (Allen and Berkley, 1979; Shinn-Cunningham et al., 2001) to generate realistic BRIRs, including a distinct direct sound, individual early reflections, and the dense superposition of late reflections. The resemblance of BRIRs created by this algorithm with acoustic measurements is supported by several studies (Allen and Berkley, 1979; Shinn-Cunningham et al., 2001; Zahorik, 2009). On the one hand, using a more realistic BRIR might have posed a more demanding task than the simplified reverberation model used by Mesgarani et al. (2014). On the other hand, binaural stimuli may hold an advantage over monaural stimuli in reverberation as a result of the IAC fluctuations (Slama and Delgutte, 2015), although, as explained above, it is hard to ascertain whether this advantage was present with our speech stimuli.

The use of different animal models in our study and that of Mesgarani et al. (2014) may have contributed to the different pattern of results with reverberant stimuli. However, ferrets (Walker et al., 2017) and rabbits (Wagner et al., 2022) use similar acoustic cues to perceptually discriminate the fundamental frequency (F0) of harmonic complex tones, which characterizes voiced speech. We believe that differences in recording sites (A1 vs IC) are likely to be more important than species differences in accounting for the different results in the two studies. Several studies have shown clear differences between the encoded information in the IC and A1 for complex stimuli such as speech. For example, Rabinowitz et al. (2013) recorded from single and multiunits in IC and A1 of anesthetized ferrets in response to natural sounds embedded in background noise. They found that neural responses were more tolerant to variations in noise level in A1 compared with IC. Chechik et al. (2006) recorded from single units in the IC, the auditory thalamus, and the A1 of anesthetized cats in response to bird vocalizations. They found that IC neurons were more redundant, in terms of the stimulus information they encoded than neurons in the auditory thalamus and A1. Chechik et al. (2006) suggest that this decrease in redundancy reflects the extraction of meaningful features in the stimuli (Barlow, 2001). Overall, these results are consistent with our observations that neural responses in rabbit IC closely followed the stimulus envelope, and that the degradation in neural coding because of reverberation paralleled degradations present in the stimulus itself. However, it remains to be seen whether future studies in rabbit A1 would yield more robust and reverberation-free reconstructions as compared with units in the midbrain.

Mesgarani et al. (2014) compared the ability of different models based on spatiotemporal receptive fields (STRF) to account for the robust neural coding of noisy and reverberant speech in the ferret A1. They found that only a model incorporating both subtractive synaptic depression and multiplicative gain normalization was able to account for the physiological results in both noise and reverberation. Since synaptic depression is common throughout the auditory pathway, including the hair cell ribbon synapse (Goutman, 2017), the cochlear nucleus (H. Yang and Xu-Friedman, 2008, 2009), and IC (Wu et al., 2002), the failure to observe robust coding of speech in reverberation in the IC may reflect the lack of a multiplicative gain normalization mechanism in the auditory midbrain or just a weaker gain normalization compared with A1.

The degradation in spectrogram reconstruction quality we observed with increasing reverberation may appear to conflict with our finding of an increase in neural modulation gain, which was interpreted as a form of robustness to reverberation. However, the increase in modulation gain just implies that the amplitude of neural modulation is relatively well preserved in reverberation. Spectrogram reconstruction quality, on the other hand, does not depend on the amplitude of neural modulation but rather on how well these modulations track the envelope of the dry stimulus.

### Sources of degradation in the linear optimal reconstruction model

To analyze the degradation in spectrogram reconstruction quality in reverberation, we aimed to isolate three factors that are likely to contribute to the degradation of the reconstruction process: envelope tracking errors, model generalization failure, and distortion compensation failure. To do so, we compared three reconstruction methods differing in the data used for training and testing the model.

Envelope tracking errors refer to the inability of the linear model trained on a given reverberant condition to reconstruct new unseen spectrograms in the same reverberant condition (Fig. 9, yellow bars). This type of error was an important contributor to the degradation in spectrogram reconstruction quality with increasing reverberation. That envelope tracking errors increase with increasing reverberation may seem surprising since the model is both trained and tested with stimuli having the same degree of reverberation. It suggests reverberant speech is intrinsically more difficult to reconstruct from neural ensemble response than dry speech. One possible explanation is that the neural signal-to-noise ratio decreases with increasing reverberation. Here, the “signal” refers to the modulations in the stimulus envelope and the resulting modulation in the neural response. The “noise” refers to variability in neural responses across different presentations of the same stimulus. Since the signal (the envelope modulations) decreases with increasing reverberation while Poisson-like neural noise is expected to stay approximately constant if the overall firing rate does not change in reverberation, the signal-to-noise ratio is likely to decrease with increasing reverberation. A decrease in the signal-to-noise ratio of the reconstructed spectrogram will result in a decrease in its correlation with the stimulus spectrogram, as observed. Unfortunately, we used too few stimulus trials (five for reverberant speech) to reliably estimate the neural noise and directly test this hypothesis.

Model generalization failure refers to the additional degradation in reconstruction quality when the linear model is trained with responses to dry speech and tested against reverberant spectrograms. This type of error also contributed to the degradation in spectrogram reconstruction quality, especially for MU responses in severe reverberation. Lastly, distortion compensation failure refers to the additional degradation in reconstruction quality when the model is trained with dry stimuli and tested by comparing spectrograms reconstructed from reverberant responses against spectrograms for dry stimuli. It indicates the model’s inability to compensate for reverberation given that it was trained on dry stimuli and thus constitutes the most specific evidence against a dereverberation mechanism. This type of error contributed mostly in severe reverberation, and for reconstructions based on multiunit responses. Overall, results suggest that all three factors contribute to the degradation in reconstruction quality with increasing reverberation, with envelope tracking error being the most important factor.

In conclusion, using single-unit and multiunit recordings from the auditory midbrain of unanesthetized Dutch-belted rabbits, we characterized the effect of various degrees of reverberation on the neural coding of natural speech utterances. We analyzed recordings of single-site and ensemble responses via optimal linear reconstruction techniques to quantify the correlation between stimuli envelopes and neural responses. Although spectrogram reconstruction quality was high for dry speech and in moderate reverberation, the neural representation of speech was degraded in severe reverberation, and thus we found no evidence for a neural compensation mechanism for the effect of reverberation in the IC of unanesthetized rabbits when studied with linear reconstruction techniques.

## Synthesis

Reviewing Editor: Christine Portfors, Washington State University Decisions are customarily a result of the Reviewing Editor and the peer reviewers coming together and discussing their recommendations until a consensus is reached. When revisions are invited, a fact-based synthesis statement explaining their decision and outlining what is needed to prepare a revision will be listed below. The following reviewer(s) agreed to reveal their identity: Mahtab Tehrani.

This study has a comprehensive approach to understanding neural processing of acoustic reverberation, a phenomenon that is important for advancing our knowledge of speech perception in humans. The choice of stimulus and reverberation paradigm allowed for a more detailed understanding of the mechanism of neural reverberation as compared to previous work. Complementary metrics were used in several analyses which added value and rigor. These analyses enabled the authors to find no evidence of dereverberation/compensation in the auditory midbrain which is a novel finding.

Minor comments:

1.Figure 6 caption, lines 8 and 9: “The median MG tended to INCREASE DECREASING...”. Grammar needs attention.

2.Line 639: “...as was HE interaction between the two factors”- change to “the”.

3.Figure 11A. Y-axis legend partially obscured by the letter “A”.

4. It would be helpful to provide some rationale for the use of human speech signals as stimuli for a rabbit and why the particular sentences were chosen.

5. It would be helpful to provide a description of the recording sites. Were the recordings biased at all in their locations the IC? Was the IC sampled thoroughly? Were the recordings focused on the central nucleus or were they in all subdivisions of the IC. Were there any differences in the responses based on location in the IC.

## Author Response

Synthesis Statement for Author (Required):

This study has a comprehensive approach to understanding neural processing of acoustic reverberation, a phenomenon that is important for advancing our knowledge of speech perception in humans. The choice of stimulus and reverberation paradigm allowed for a more detailed understanding of the mechanism of neural reverberation as compared to previous work. Complementary metrics were used in several analyses which added value and rigor. These analyses enabled the authors to find no evidence of dereverberation/compensation in the auditory midbrain which is a novel finding.

We thank the reviewers and the reviewing editor for their critique of this rather complicated

paper.

Minor comments:

1.Figure 6 caption, lines 8 and 9: “The median MG tended to INCREASE DECREASING...”.

Grammar needs attention.

This has been corrected.

2.Line 639: “...as was HE interaction between the two factors”- change to “the”.

This has been corrected.

3.Figure 11A. Y-axis legend partially obscured by the letter “A”.

This has been corrected.

In addition to these corrections, we have made a number of minor changes throughout the paper to fix additional typos and improve the clarity of writing
